# Breast cancer risk and genetic ancestry: a case–control study in Uruguay

**DOI:** 10.1186/s12905-015-0171-8

**Published:** 2015-02-18

**Authors:** Carolina Bonilla, Bernardo Bertoni, Pedro C Hidalgo, Nora Artagaveytia, Elizabeth Ackermann, Isabel Barreto, Paula Cancela, Mónica Cappetta, Ana Egaña, Gonzalo Figueiro, Silvina Heinzen, Stanley Hooker, Estela Román, Mónica Sans, Rick A Kittles

**Affiliations:** School of Social and Community Medicine, University of Bristol, Oakfield House, Oakfield Grove, Bristol, BS8 2BN UK; Departamento de Genética, Facultad de Medicina, Universidad de la República, Montevideo, Uruguay; Polo de Desarrollo Universitario “Variabilidad Genética Humana”, Centro Universitario de Tacuarembó, Universidad de la República, Tacuarembó, Uruguay; Departamento de Antropología Biológica, Instituto de Ciencias Antropológicas, Facultad de Humanidades y Ciencias de la Educación, Universidad de la República, Montevideo, Uruguay; Departamento Básico de Medicina, Hospital de Clínicas, Facultad de Medicina, Universidad de la República, Montevideo, Uruguay; Laboratorio de Oncología Básica y Biología Molecular (LOBBM), Facultad de Medicina, Universidad de la República, Montevideo, Uruguay; Polo de Desarrollo Universitario “Centro de investigaciones interdisciplinarias sobre la presencia indígena misionera en el territorio: patrimonio, región y fronteras culturales”, Centro Universitario de Tacuarembó, Universidad de la República, Tacuarembó, Uruguay; Unidad Académica de la Licenciatura en Biología Humana, Centro Universitario de Paysandú, Universidad de la República, Paysandú, Uruguay; Section of Genetic Medicine, Department of Medicine, Pritzker School of Medicine, University of Chicago, Chicago, Illinois USA; Department of Medicine, Section of Hematology and Oncology, University of Illinois at Chicago, Chicago, Illinois USA; Present address: Department of Molecular and Human Genetics, Baylor College of Medicine, Houston, Texas USA; Present address: Center for Population Genetics, The University of Arizona College of Medicine, Tucson, Arizona USA

**Keywords:** Breast cancer, Population admixture, Ancestry informative markers, Mitochondrial haplogroups, Latin America, Uruguay

## Abstract

**Background:**

Uruguay exhibits one of the highest rates of breast cancer in Latin America, similar to those of developed nations, the reasons for which are not completely understood. In this study we investigated the effect that ancestral background has on breast cancer susceptibility among Uruguayan women.

**Methods:**

We carried out a case–control study of 328 (164 cases, 164 controls) women enrolled in public hospitals and private clinics across the country. We estimated ancestral proportions using a panel of nuclear and mitochondrial ancestry informative markers (AIMs) and tested their association with breast cancer risk.

**Results:**

Nuclear individual ancestry in cases was (mean ± SD) 9.8 ± 7.6% African, 13.2 ± 10.2% Native American and 77.1 ± 13.1% European, and in controls 9.1 ± 7.5% African, 14.7 ± 11.2% Native American and 76.2 ± 14.2% European. There was no evidence of a difference in nuclear or mitochondrial ancestry between cases and controls. However, European mitochondrial haplogroup H was associated with breast cancer (OR = 2.0; 95% CI 1.1, 3.5).

**Conclusions:**

We have not found evidence that overall genetic ancestry differs between breast cancer patients and controls in Uruguay but we detected an association of the disease with a European mitochondrial lineage, which warrants further investigation.

**Electronic supplementary material:**

The online version of this article (doi:10.1186/s12905-015-0171-8) contains supplementary material, which is available to authorized users.

## Background

Breast cancer is the most common malignancy among women, with an annual incidence worldwide of one million cases, of which almost 60% occur in the United States and Europe [1]. The distribution of incidence and mortality rates varies between countries and even between populations within a country. Although South America is considered a low risk region [1] there are important differences in breast cancer rates among countries across the continent (globocan.iarc.fr). Notably, the temperate region of South America, which includes Argentina, Chile, Uruguay and southern Brazil exhibits rates comparable to those of North America, Australia, New Zealand and some areas in Europe. In the United States annual age-adjusted incidence rates have been reported to be higher in European American women (127/100,000) than in African American (118/100,000), Hispanic (91/100,000), American Indian/Alaskan Native (90/100,000) and Asian/Pacific islander (85/100,000) women [2]. However, African American females exhibit higher breast cancer mortality, are likely to be diagnosed at a more advanced stage and present larger tumors than their European American counterparts [3]. The disparities observed between and within regions and populations are unclear but may be attributed to differences in environmental and/or genetic exposures [1].

Among Uruguayan women breast cancer is the most frequent cancer type as well as being the most common cause of death related to cancer. The nation’s overall age-adjusted incidence rate is 71 cases per 100,000 women per year, and mortality rates due to breast cancer reach 23 per 100,000 women per year [4,5]. These rates are the highest in Latin America and are close to the rates observed in Western developed countries. The reasons for such high frequency of breast cancer in Uruguay are not well-understood, but a number of breast cancer risk factors occur frequently in the Uruguayan population. Increased consumption of red meat and fat, as well as a reduced intake of vegetables have been identified as risk factors for breast cancer in Uruguay [6-8]. Even though the association of lifestyle conditions, especially diet, with breast cancer risk has been thoroughly investigated [6-11], with few exceptions, the effect that genetic risk factors may have on the prevalence of breast cancer in this population has not been comprehensively assessed so far [12-14].

Earlier studies have shown that European genetic ancestry estimated using a set of nuclear ancestry informative markers (AIMs) is positively associated with breast cancer risk in US Hispanic and Mexican women [15,16]. The non-European genetic contribution to the population of Uruguay has been estimated as ~10% Native American and ~6% African [17]. But maternal lineages assessed using mitochondrial DNA (mtDNA) revealed a Native American ancestral proportion of 62% in the north and 20% in the south (mean value for the country ~ 34%), while the African contribution varies between 8% and 21% [18-22].

In this study we investigated the association of ancestry with breast cancer risk in Uruguayan women, using nuclear and mtDNA AIMs to extensively characterize the ancestral background of patients and controls.

## Methods

### Study population

A sample of 200 sporadic breast cancer patients and 216 controls was collected in public hospitals and private clinics across Uruguay. Women younger than 45 years of age or with more than one affected relative were excluded from participation to prevent the inclusion in the study of familial cases. The health centers where enrollment took place were Hospital de Clínicas “Manuel Quintela”, Centro Hospitalario Pereira Rossell, Hospital Central de las Fuerzas Armadas, Instituto Nacional del Cáncer and Casa de Galicia in Montevideo (the capital, southern region), and four hospitals in other regions, i.e. Asistencial de San Carlos (Maldonado, southeast), Hospital de Tacuarembó (Tacuarembó, northeast), Cooperativa Médica de Paysandú (Paysandú, northwest) and Hospital de Soriano (Soriano, southwest). All patients had been diagnosed with breast cancer a year or less before inclusion in the study. Controls were selected from the same hospitals and clinics as the patients, and similarly, control women with a family history of breast cancer were excluded. Patients and controls filled in a questionnaire that gathered information on socio-demographic, lifestyle, reproductive and family history variables. Individuals recruited from centers where only cases or only controls were available were excluded from analysis. Thus, out of the 416 individuals enrolled to the study, 328 (164 cases and 164 controls) were further analyzed. Of these 141 cases and 139 controls were recruited in public hospitals, whereas 23 cases and 25 controls were recruited in private clinics. A descriptive summary of the study population, by disease status, is given in Table 1.

**Table 1:** **Characteristics of the study population**

	**Cases**	**Controls**	**p-value**
**Age at diagnosis/recruitment (years)**			
mean ± SD	56.6 ± 10.1	52.8 ± 8.9	<0.001
**Place of residence (%)**			
Montevideo	86.5	87.2	0.85
Outside Montevideo	13.5	12.8	
**Location (%)**			
Urban^a^	85.5	90.6	0.17
Rural	14.5	9.4	
**Hospital (%)**			
Public	87.2	84.8	0.54
Private	12.8	15.2	
**Age at menarche (years)** ^**f**^			
mean ± SD	12.3 ± 1.5	12.2 ± 1.4	0.56
**Oral contraceptives (%)**			
Yes	66.2	73.8	0.15
No	33.8	26.2	
**Children (%)** ^**t**^			
Yes	90.5	91.5	0.78
No	9.5	8.5	
**Age at first child (years)** ^**j**^			
mean ± SD	25.6 ± 6.0	23.4 ± 6.0	0.01
**Number of children (%)** ^**i**^			
mean ± SD	2.46 ± 1.52	2.52 ± 1.73	0.75
**Breastfeeding (%)** ^**k**^			
Yes	80.2	85.5	0.24
No	19.8	14.5	
**Menopause** ^**g,r**^ **(%)**			
Yes	65.2	52.1	0.03
No	34.9	47.9	
**Age at menopause (years)** ^**h,s**^			
mean ± SD	49.2 ± 5.0	47.6 ± 4.3	0.07
**Breast cancer in the family (%)**			
Yes	18.2	9.8	0.03
No	81.8	90.2	
**Smoking (%)** ^**l**^			
Yes	47.3	53.4	0.28
No	52.7	46.6	
**Passive smoker before 20 years old (%)** ^**m**^			
Yes	55.4	54.0	0.81
No	44.6	46.0	
**Red meat intake** ^**c**^ **(%)** ^**n,u**^			
Yes	95.1	100.0	0.01^v^
No	4.9	0.0	
**Education (%)**			
Primary school	37.8	32.3	0.001
Secondary school	53.3	43.3	
University	8.9	24.4	
**Occupation (%)** ^**o**^			
Unemployed/never worked	9.6	0.6	<0.001^v^
Self-employed	84.3	70.0	
Public/private employee	3.4	10.0	
Teacher	2.7	4.4	
Retired	0.0	4.4	
Professional	0.0	10.6	
**Income (U$S per month, %)** ^**p**^			
<175	6.1	7.0	<0.001
175-750	87.0	66.7	
>750	6.9	26.3	
**Source of income (%)** ^**q**^			
Social security/family	12.2	3.7	<0.001^v^
Pension	20.9	4.4	
Salary	64.2	88.8	
Rent	2.7	1.2	
Other	0.0	1.9	
**Socioeconomic status (%)**			
Low	38.5	32.3	0.25
Medium	61.5	67.7	
**Weight (kg)** ^**b**^			
mean ± SD	71.2 ± 14.9	68.5 ± 13.5	0.11
**Height (m)** ^**c**^			
mean ± SD	1.60 ± 0.07	1.59 ± 0.07	0.31
**BMI (kg/m** ^**2**^ **)** ^**d**^			
mean ± SD	27.7 ± 5.3	27.0 ± 5.1	0.22
**Waist-hip ratio** ^**e**^			
mean ± SD	0.87 ± 0.07	0.86 ± 0.08	0.17
**Ancestry (%) mean ± SD**			
African	9.8 ± 7.6	9.1 ± 7.5	0.33
Native American	13.2 ± 10.2	14.7 ± 11.2	0.26
European	77.1 ± 13.1	76.2 ± 14.2	0.73

N cases = 148; N controls = 164.

Missing data (N cases/N controls): ^a,b^3/4, ^c^10/3, ^d^10/4, ^e^16/29, ^f^0/3, ^g^16/22, ^h^82/115, ^i,m^27/12, ^j^39/25, ^k^27/28, ^l^2/3, ^n^6/12, ^o^2/4, ^p^2/8, ^q^0/3.

^r^Women who underwent menopause due to treatment or surgical intervention were excluded. Not all participants who answered this question also responded the question on age at menopause, thus the difference in sample sizes.

^s^Age at menopause was calculated for women who were ≥ 55 years old and had undergone menopause naturally.

^t^Not all participants who answered this question also responded the question on age at first child, thus the difference in sample sizes.

^u^Among cases, red meat intake corresponds to frequency of intake before being diagnosed with breast cancer.

^v^Fisher’s exact test.

This study was approved by the University of Chicago and the Universidad de la República review boards and all individuals provided written informed consent to participate.

### DNA extraction

Genomic DNA was extracted from peripheral blood leukocytes using a FlexiGene® DNA Kit (QIAGEN) and stored at −20°C until analysis. SNP genotyping was undertaken by KBioscience Ltd. (www.kbioscience.co.uk), who use their own form of competitive allele specific PCR system (KASPar), and also at the University of Chicago using Sequenom MassArray technology (Sequenom Inc., San Diego, CA, USA) [23].

### Genetic analyses

#### Nuclear ancestry informative markers

We genotyped 166 AIMs located along all autosomes and the X chromosome in 400 individuals for which DNA was successfully extracted (189 cases and 211 controls). These were part of two sets of AIMs: 109 were previously defined by us [24], and 57 were obtained from a panel published by Fejerman et al. [16].

AIMs with minor allele frequencies below 1% and genotyping rate lower than 90% were excluded. After filtering there remained 160 AIMs for ancestry estimation (Additional file 1: Table S1).

Seventy-two individuals were genotyped but later excluded from analysis because they were recruited in centers where only cases or only controls were available (see above). Complete genotyping to estimate nuclear ancestral proportions was successful in 312 participants (148 cases and 164 controls).

The parental populations used to estimate admixture proportions included 42 Europeans (Coriell’s North American panel), 37 West Africans (non-admixed Africans living in London, UK, and South Carolina, USA), and 30 Native Americans (15 Mayans and 15 Nahuas), which were genotyped on an Affymetrix 100 K SNP chip (data kindly provided by Dr. Laura Fejerman, UCSF). Additionally we had AIM genotypes for 243 Europeans (from England, Germany, Ireland and Spain), 279 Africans (from Central African Republic, Nigeria and Sierra Leone), and 184 Native Americans (Cheyenne, Maya, Pima, and Pueblo).

We calculated individual African, Native American and European ancestry in affected and unaffected women using the program Structure [25]. Given the tri-hybrid parental contributions to the Uruguayan population, described in earlier studies (see [26] for a review), the program was run mainly with K = 3, but also with K = 2 due to the African contribution being somewhat low, as the predefined setting for the number of ancestral populations, with 10,000 iterations for the burn-in period and 50,000 additional iterations to obtain parameter estimates. In all cases the program was instructed to use parental population information. Several options were explored, such as the admixture and linkage models, and independent or correlated allele frequencies, to identify changes in the clustering pattern.

#### Mitochondrial DNA ancestry informative markers

All 328 samples were successfully genotyped and/or sequenced and assigned to mtDNA haplogroups. We analyzed 14 mtDNA restriction sites that define continental haplogroups and sequenced the hypervariable region I (HVRI, from 16025 to 16569 bp). Individuals were initially assayed for polymorphic variants characterizing major Native American haplogroups A, B, C and D [27] (Additional file 1: Table S2). If these polymorphisms were absent the presence of restriction site 7025, which defines European haplogroup H, was investigated. Subjects not carrying any of the above were tested for other specific restriction sites for haplogroup identification based on the HVRI sequence. The HVRI was amplified using primers 15996 F (5’-CACCATTAGCACCCAAAGCT-3’ [28]) and 16011R (5’-CGTGAGTGGTTAATAGGGTGATAG-3’; designed by GF). In cases with uncertain haplogroup assignments HVRII was sequenced using primers 29 F (5’-GGTCTATCACCCTATTAACCAC-3’ [28]) and 397R (5’-CATACCGCCAAAAGATAAAAT-3’ [29]). PCR amplification conditions were set according to Martinez-Cruzado et al. [30], with minor modifications. An initial denaturation step was performed at 95°C for 5 minutes, annealing and extension conditions varied depending on the polymorphism (provided on request). The amplification products were checked by electrophoresis on a 2% agarose gel with ethidium bromide staining. PCR products for haplogroup assignment (with the exception of haplogroup B) were digested overnight with one unit of the appropriate restriction enzyme and detected using polyacrylamide gel electrophoresis (8% T, 3% C) with silver staining. In the case of haplogroup B, which is an insertion/deletion polymorphism, no digestion was necessary and PCR products were visualized as described above.

PCR products for sequencing were purified using silica spin columns. Sequencing was carried out by an external service provider (Macrogen Inc., Seoul, South Korea) and at the Institut Pasteur Montevideo (Uruguay). Sequences were examined using the Chromas 2.01 software (Technelysium Pty Ltd.) and aligned using the Genedoc software version 2.7.000 [31].

Haplogroup definition was established following published criteria [32-38], and using the mtDNA Manager tool [39] and Phylotree (mtDNA tree Build 15) [38].

### Statistical analyses

Assessment of the association of non-genetic risk factors with susceptibility to breast cancer was carried out using t-tests, ANOVA, and chi-square tests.

Differences in ancestry estimates between cases and controls were examined with the Wilcoxon rank-sum test. The association of ancestry with confounders was assessed using linear regression in the case of age, and the continuous anthropometric and reproductive variables, and Kruskal-Wallis and Wilcoxon rank-sum tests in the case of categorical variables. Chi-square tests were applied to compare mtDNA haplogroup distributions. Multivariable logistic regressions were used in the analysis of nuclear and mitochondrial ancestry and disease with adjustment for age, educational achievement and hospital where recruitment took place. Other potential confounders were not included in the models because of sample size reductions due to missing data. All analyses were carried out using the statistical package Stata (StataCorp, 2012, College Station, TX).

Hardy-Weinberg equilibrium was ascertained for all nuclear AIMs using the program PLINK [40]. The association of individual AIMs with breast cancer was examined using the Cochran-Armitage trend test and multivariable logistic regression with additive SNP effects implemented in PLINK.

## Results

### Characteristics of the study population

Patients were on average older, less likely to have a university education, and more likely to be unemployed, have lower income and depend more on social security and a pension than controls. Additionally, patients were more likely to have relatives with breast cancer. The mean age at diagnosis was 57 years old, the majority of affected women being postmenopausal (65% compared to 52% in controls). Other well-known breast cancer risk factors, such as early age at menarche, taking oral contraceptives, nulliparity, and breastfeeding, did not show evidence of association with the disease. Age at birth of first child and at menopause was higher in cases than in controls. Among controls, red meat consumption was slightly more frequent than in patients (Table 1).

### Ancestry and breast cancer risk

#### Nuclear DNA ancestry

AIMs rs7504, rs35395 and rs1341567 were out of Hardy-Weinberg equilibrium (p < 0.001), after a Bonferroni correction for multiple testing (data available on request).

**Figure 1:**
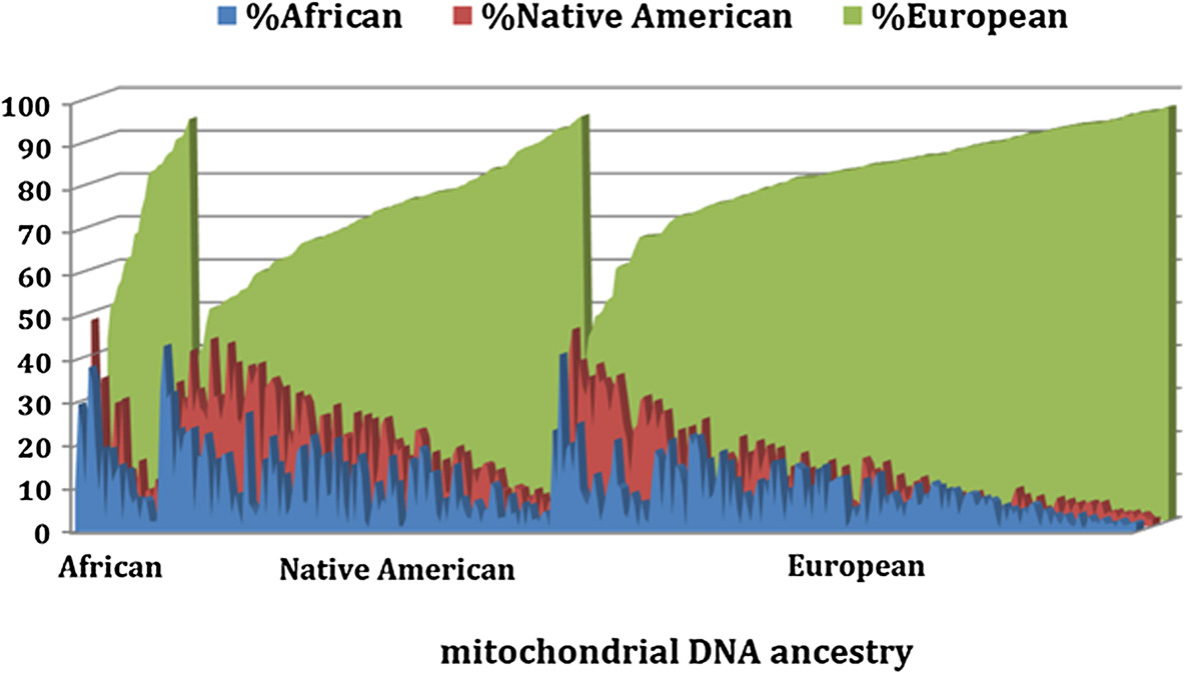
**Ancestral proportions in Uruguayan women (cases and controls, N = 312).** Each vertical bar along the x-axis represents an individual, bars are shaded according to an individual’s nuclear genetic ancestry, and individuals are grouped according to their maternal ancestry.

We confirmed the tri-hybrid nature of the Uruguayan population, which showed contributions from Africans, Native Americans and Europeans. We found that the Structure model assuming a three-way admixture process provided the best fit, compared to models considering either one or two parental populations (data not shown). Ancestral proportions in the population as a whole were (mean ± SD) 9.4 ± 7.5%, 14.0 ± 10.8% and 76.6 ± 13.7%, respectively. There was considerable variation in ancestry among participants. Nuclear DNA African ancestry ranged from 1% to 43%, Native American ancestry from 1% to 48%, and European ancestry from 33% to 96% (Figure 1). Ancestry estimates for patients and controls are shown in Table 1. There was no evidence of a difference in ancestry between cases and controls.

Among unaffected individuals we found variations in ancestry with educational attainment, socioeconomic status (including occupation and income) and hospital where receiving care, but not with place of residence or location (Table 2, Additional file 1: Table S3). Participants who attended university exhibited more European ancestry and less Native American and African ancestry than those who had finished secondary school or primary school only. Similarly, professionals and women who earned over U$S 750 per month, had higher levels of European ancestry, with concomitantly lower levels of Native American and African ancestry (Additional file 1: Table S3). In addition, Native American ancestry was inversely associated with height, age at first child and age at menopause, and positively with BMI, whilst European ancestry was positively associated with age at first child and at menopause. There was also some indication that age at menopause was inversely associated with African ancestry, and that pre-menopausal women had higher African and lower European ancestry (Table 2 and Additional file 1: Table S3). Age was not associated with ancestry.

**Table 2:** **Potential confounders and nuclear individual ancestry in Uruguayan controls**

	**% African (mean ± SD)**	**% Native American (mean ± SD)**	**% European (mean ± SD)**
**Controls (N = 164)**			
**Place of residence (N)**			
Montevideo (143)	9.3 ± 7.8	15.3 ± 11.6	75.4 ± 14.7
Rest of the country (21)	8.1 ± 5.2	10.5 ± 7.2	81.4 ± 7.8
p-value	0.81	0.13	0.16
**Hospital (N)**			
Public (139)	9.5 ± 7.8	15.5 ± 11.7	75.1 ± 14.8
Private (25)	7.4 ± 5.4	10.2 ± 7.0	82.4 ± 6.8
p-value	0.27	0.07	0.05
**Menopause (N)**			
Yes (91)	8.6 ± 7.6	13.9 ± 10.7	77.5 ± 14.6
No (69)	10.2 ± 7.4	16.1 ± 12.0	73.7 ± 13.4
p-value	0.05	0.32	0.04
**Breast cancer in the family (N)**			
Yes (16)	8.6 ± 5.9	16.0 ± 12.6	75.4 ± 15.0
No (148)	9.2 ± 7.6	14.5 ± 11.1	76.3 ± 14.1
p-value	0.78	0.70	0.81
**Education (N)**			
Primary school (53)	10.0 ± 8.1	17.6 ± 12.3	72.5 ± 16.0
Secondary school (71)	9.9 ± 7.9	15.4 ± 10.9	74.7 ± 13.1
University (40)	6.7 ± 5.1	9.5 ± 8.5	83.8 ± 10.2
p-value	0.08	0.001	<0.001
**Socioeconomic status (N)**			
Low (53)	9.9 ± 8.1	16.7 ± 11.7	73.4 ± 15.6
Medium (111)	8.8 ± 7.2	13.7 ± 10.9	77.5 ± 13.3
p-value	0.41	0.07	0.13
**Age at recruitment** (mean change in years per 25% ancestry increase, 95% CI) (N = 164)	−0.56 (−1.75, 0.63)	0.08 (−1.17, 1.33)	0.52 (−0.68, 1.73)
p-value	0.36	0.90	0.39
**Age at first child** (mean change in years per 25% ancestry increase, 95% CI) (N = 139)	−0.44 (−1.26, 0.39)	−0.91 (−1.78, −0.03)	0.94 (0.11, 1.78)
p-value	0.30	0.04	0.03
**Age at menopause** (mean change in years per 25% ancestry increase, 95% CI) (N = 49)^a^	−0.94 (−1.90, 0.02)	−1.60 (−2.66, −0.54)	1.43 (0.46, 2.40)
p-value	0.05	0.004	0.01

^a^Age at menopause was calculated for women who were ≥ 55 years old and had undergone menopause naturally.

The association of ancestry with some of the reproductive variables examined here, which are well-known breast cancer risk factors, suggests that they could mediate a potential association of ancestry with breast cancer, however these variables were also strongly associated with socioeconomic status and education (data not shown).

We ran logistic regression models to test for the effect of ancestry on breast cancer risk with adjustment for age, education and hospital. Education was strongly correlated with socioeconomic status, therefore we did not include the latter in the analyses (correlation coefficient = 0.71, p < 0.0001). European ancestry was positively associated with risk, as was African ancestry, whereas Native American ancestry showed an inverse association. However, there was no strong statistical support for these effects (Table 3).

**Table 3:** **Association of nuclear and mitochondrial ancestry with breast cancer risk in Uruguayan women**

**Nuclear ancestry**	**OR** ^**a**^	**95% CI**	**p-value**
European	1.05	(0.85,1.30)	0.63
African	1.09	(0.89,1.33)	0.42
Native American	0.84	(0.68,1.04)	0.10
N	312		
**Mitochondrial ancestry**	**OR** ^**b**^	**95% CI**	**p-value**
European	Reference		0.25^c^
African	0.54	(0.22,1.33)	0.18
Native American	0.71	(0.42,1.20)	0.20
N	312		
No H haplogroup	Reference		
H haplogroup	1.99	(1.13,3.51)	0.02
N	312		
No H haplogroup	Reference		
H haplogroup	1.84	(0.97,3.51)	0.06
N^d^	171		

All regression models were adjusted for age, education and hospital. Mitochondrial ancestry regression models were additionally adjusted for European nuclear ancestry.

^a^Breast cancer OR per 25% increase in ancestry.

^b^Breast cancer OR for women carrying an African or Native American mitochondrial haplogroup compared to women carrying a European haplogroup, or women carrying a H haplogroup compared to those carrying a different haplogroup.

^c^P-value for the model.

^d^Analysis restricted to women carrying a European mitochondrial haplogroup.

There were 16 AIMs associated with breast cancer risk as indicated by the Cochran-Armitage trend test (p ≤ 0.05). Thirteen AIMs showed an association with disease when adjustment for European and Native American ancestry was performed (Additional file 1: Tables S4 and S5). The AIM eliciting the strongest association with breast cancer was rs10486576 on 7p15 within locus *JAZF1* (per allele OR = 2.0; 95% CI 1.2, 3.1; p = 0.01). No associations were detected if a correction for multiple testing was implemented.

#### Mitochondrial DNA ancestry

The analysis of mtDNA revealed that the contributions from the three parental groups were 7.6% African, 36.7% Native American and 55.7% European. Among controls 9.8% of individuals carried an African haplogroup, 39.6% a Native American haplogroup, and 50.6% a European haplogroup, whereas among cases the respective figures were 5.5%, 33.7% and 60.8%. Case and control groups were not markedly different from each other (p = 0.12).

Potential confounders were not associated with mtDNA ancestry with the exception of hospital and place of residence, and weakly with income, in controls. Individuals with a Native American mitochondrial haplogroup were less likely to receive medical attention at a private clinic, and to reside outside Montevideo (Additional file 1: Table S6).

Women with maternal Native American or African ancestry were at a lower risk of developing breast cancer than women with maternal European ancestry but statistical support for this finding was lacking (Table 3).

The distribution of mtDNA haplogroups in patients and controls is shown in Table 4. There was no evidence of a differential haplogroup distribution between affected and unaffected subjects. However, when we examined haplogroup H exclusively we found an association with breast cancer, overall and in participants who carried a European mtDNA lineage, after adjustment for age, education, hospital and nuclear European ancestry (Table 3).

**Table 4:** **Mitochondrial DNA haplogroups in breast cancer cases and controls from Uruguay**

**Haplogroup**	**Cases**	**Controls**	**p-value**
African	9	16	
L	9	16	
Native American	55	65	0.40^a^
A	11	19	
B	21	16	
C	16	21	
D	7	9	
European	99	83	0.26^a^
H	48	27	
HV	1	3	
I	0	1	
J	9	5	
K	4	8	
N	1	1	
R	0	2	
T	9	11	
U1-5	8	8	
U6	12	9	
V	2	0	
W	3	6	
X	2	2	
Total	163^d^	164	0.20^b^/0.12^c^

^a^P-value corresponding to the Pearson’s X^2^ statistic obtained when examining differences in haplogroup distribution between cases and controls within each ancestry.

^b^P-value obtained from testing the distribution of all mtDNA haplogroups in cases and controls using Pearson’s X^2^ test.

^c^P-value obtained from testing the Native American, African and European ancestral contributions in cases and controls using Pearson’s X^2^ test.

^d^An individual carrying a G haplogroup, which is of Asian origin, was not included in the analysis.

## Discussion

Our study has uncovered evidence of population stratification in Uruguay with likely roots in an admixture process that involved parental African, Native American and European populations. Ancestry estimates obtained for this population sample are comparable to previously published nuclear and mitochondrial estimates for Uruguay [17-22]. Population structure was evident in the inter-individual variation in admixture proportions, the admixture LD between unlinked AIMs (data not shown), and the excess association of AIMs with disease (over the expected 5% positive results).

The strongest associated AIM, although not showing a robust enough association to overcome a Bonferroni correction for multiple testing, rs10486576, is located in the *JAZF1* locus, a gene implicated in type 2 diabetes (T2D) [41], height [42] and prostate cancer [43]. Two separate regions have been identified within *JAZF1* that independently explain the associations with each disease [44]. In fact, variant rs10486567 in this gene has been associated with prostate cancer but not with T2D. Whether this is a true breast cancer signal in this population remains to be established.

We did not find strong evidence of association of nuclear and mitochondrial ancestry estimates with breast cancer risk. This may reflect a lack of statistical power, consequence of a small sample size. On the other hand, it may be indicative of the role of non-genetic factors, possibly related to lifestyle and environmental conditions, on the onset of breast cancer. Epigenetic effects may also be at play (Cappetta et al., submitted). Although not robust, we observed an increased risk for breast cancer with higher European ancestry and, concurrently, a protective effect of Native American ancestry. The direction of the ancestry effects we uncovered is consistent with associations reported for US Latinas and Mexican women [15,16,45] for which a higher nuclear European/lower Native American ancestral contribution increased breast cancer susceptibility. The diverse ancestral proportions, and different parental sources, of Uruguayans with respect to those of Mexicans and US Hispanics may have also influenced our ability to detect an effect of ancestry in this population.

Nuclear ancestral proportions varied with educational achievement, different indicators of socioeconomic status and hospital where receiving medical attention. Individuals who attended university, reported being of middle class status and were recruited at a private clinic had on average higher European and lower African and Native American ancestry than participants of a lower socioeconomic position. Age at first child and age at menopause were strongly associated with ancestry and at the same time with socioeconomic status and education.

Mitochondrial European haplogroup H showed an association with breast cancer susceptibility, with carriers of the haplogroup having approximately twice the risk of being diagnosed with the disease than non-carriers, whether considering all volunteers or only those of European mitochondrial ancestry. These results suggest a potential influence of ancestry on breast cancer risk that is more obvious with mitochondrial than with nuclear DNA polymorphisms, an actual effect of haplogroup H-linked variants on the disease, or on the other hand, they could be due to chance or inadequate adjustment for population stratification, and therefore require further investigation [46].

Earlier studies on the relationship of mitochondrial haplogroups and breast cancer reported an increased risk associated with haplogroups I and K and a protective role for haplogroups H and U [47-49]. These studies, however, were carried out in populations of predominantly European descent and thus, their findings may not be applicable to populations of mixed ancestry with non-European contributions or could be false positives due to underlying population stratification that was not accounted for [46,50]. In Latin America, as far as we know, the only study to date that examined the association of mitochondrial ancestry and breast cancer found a lower frequency of Native American lineages in a group of breast/ovarian cancer Chilean families with respect to the general population [51]. It was recently shown that haplogroup H was more frequent in *BRCA2* carriers whereas haplogroup X was more frequent in *BRCA1* carriers compared to patients not carrying *BRCA* mutations among Italian familial breast cancer cases [52]. Although we were careful not to recruit familial cases for our study it is interesting to note that in Uruguay there is a predominance of *BRCA2* over *BRCA1* mutations in breast cancer families, which may be linked to their ethnic origin [14].

The presence of population structure has important consequences for association studies of genetic risk factors underlying complex diseases and traits with a heterogeneous worldwide distribution as it may lead to false positive findings. Confounding by population stratification is particularly relevant in studies that examine mtDNA variants, as mtDNA is highly structured in human populations due to its lower effective population size [53].

We have shown that in the Uruguayan population the genetic ancestral background is correlated with socioeconomic status and educational attainment, a feature shared with other Latin American populations [54,55], and which is likely to confound the association between ancestry and disease [56,57]. Therefore, a word of caution is deemed necessary when conducting association studies in admixed populations, like the Uruguayan, with marked genetic heterogeneity and socioeconomic disparities.

As the characteristics of our study sample diverge in part from those of previously published studies from Uruguay (see for instance, [11]) we cannot completely rule out that the differences between cases and controls with respect to socioeconomic and reproductive factors may be a consequence of the recruitment process, which would have obscured the relationship between disease status and ancestry. Adjustment for educational level and hospital was performed in the regression models to account for this possibility. We were underpowered to carry out stratification analyses and tests of interaction.

Additionally, it is possible that, by excluding cases and controls with a family history of breast cancer, and assuming that women with such history are more likely to be of European origin [16,58], the ancestry differences between affected and unaffected individuals may have been reduced. Nonetheless, we found no differences in the ancestral proportions of cases (data not shown) or controls who reported having breast cancer in the family compared to those who did not (Table 2).

## Conclusions

We did not find differences in biparental or maternal ancestral proportions between breast cancer patients and controls in Uruguay, but we uncovered an association of European mitochondrial haplogroup H with breast cancer risk. These findings require replication in an independent sample. In addition, we note that the presence of population structure in Uruguay, likely emerging from the admixture process that took place due to the conquest and colonization of the Americas, could affect the outcome of association studies carried out in this population.

## Additional file

Additional file 1: Table S1. Ancestry informative markers (AIMs) used in the estimation of ancestral contributions to Uruguayan women. **Table S2:** Primers and restriction sites used for mitochondrial DNA haplogroup assignment. **Table S3:** Potential confounders and nuclear individual ancestry in Uruguayan controls. **Table S4:** Association of ancestry informative markers with breast cancer risk (Cochran-Armitage test). **Table S5:** Association of ancestry informative markers with breast cancer risk (logistic regression). **Table S6:** Potential confounders and mitochondrial DNA ancestry in Uruguayan controls.

## Abbreviations

AIMs: Ancestry informative markers
mtDNA: Mitochondrial DNA
HVRI: Hypervariable region I
HVRII: Hypervariable region II
