# Supplementary material for: Breast cancer risk and genetic ancestry: a case–control study in Uruguay
Source: BMC Womens Health. 2015 Feb 18;15:11. doi: 10.1186/s12905-015-0171-8 (PMC4341228; doi:10.1186/s12905-015-0171-8)
Supplement: Additional file 1: Table S1. — Ancestry informative markers (AIMs) used in the estimation of ancestral contributions to Uruguayan women. Table S2: Primers and restriction sites used for mitochondrial DNA haplogroup assignment. Table S3: Potential confounders and nuclear individual ancestry in Uruguayan controls. Table S4: Association of ancestry informative markers with breast cancer risk (Cochran-Armitage test). Table S5: Association of ancestry informative markers with breast cancer risk (logistic regression). Table S6: Potential confounders and mitochondrial DNA ancestry in Uruguayan controls. [file 12905_2015_171_MOESM1_ESM.docx]

**Additional file 1**

**Table S1.** Ancestry informative markers (AIMs) used in the estimation of ancestral contributions to Uruguayan women.

| **SNP** | **chr** | **chr position (bp)^a^** | **gene^b^** | **major/minor alleles** | **Native American^c^** | **African^c^** | **European^c^** |
| --- | --- | --- | --- | --- | --- | --- | --- |
| rs424436 | 1 | 8082678 | [ERRFI1](http://www.ncbi.nlm.nih.gov/entrez/query.fcgi?db=gene&cmd=Retrieve&dopt=Graphics&list_uids=54206) | A/G | 0.44 | 0.04 | 0.00 |
| rs2817611 | 1 | 11613163 |  | G/A | 0.04 | 0.73 | 0.05 |
| rs7504 | 1 | 27238150 | [NR0B2](http://www.ncbi.nlm.nih.gov/entrez/query.fcgi?db=gene&cmd=Retrieve&dopt=Graphics&list_uids=8431) | G/A | 0.97 | 0.40 | 0.19 |
| rs6684063 | 1 | 30699340 |  | A/C | 0.84 | 0.78 | 0.17 |
| rs1931059 | 1 | 35366056 | [DLGAP3](http://www.ncbi.nlm.nih.gov/entrez/query.fcgi?db=gene&cmd=Retrieve&dopt=Graphics&list_uids=58512) | G/A | 0.86 | 0.10 | 0.22 |
| rs10908316 | 1 | 35494810 | ZMYM6 | A/C | 0.38 | 0.91 | 0.02 |
| rs2791966 | 1 | 36314861 | [EIF2C4](http://www.ncbi.nlm.nih.gov/entrez/query.fcgi?db=gene&cmd=Retrieve&dopt=Graphics&list_uids=192670) | G/A | 0.61 | 0.85 | 0.03 |
| rs710232 | 1 | 42203154 | [HIVEP3](http://www.ncbi.nlm.nih.gov/entrez/query.fcgi?db=gene&cmd=Retrieve&dopt=Graphics&list_uids=59269) | T/G | 0.17 | 0.76 | 0.00 |
| rs1934393 | 1 | 49208618 | AGBL4, BEND5 | C/G | 0.70 | 0.78 | 0.16 |
| rs596985 | 1 | 64277035 | [ROR1](http://www.ncbi.nlm.nih.gov/entrez/query.fcgi?db=gene&cmd=Retrieve&dopt=Graphics&list_uids=4919) | A/G | 0.01 | 0.77 | 0.02 |
| rs855833 | 1 | 64357432 | [ROR1](http://www.ncbi.nlm.nih.gov/entrez/query.fcgi?db=gene&cmd=Retrieve&dopt=Graphics&list_uids=4919) | T/G | 0.01 | 0.89 | 0.11 |
| rs3828121 | 1 | 82422200 | LPHN2 | A/G | 0.70 | 0.00 | 0.13 |
| rs17035850 | 1 | 117032022 |  | A/T | 0.00 | 0.86 | 0.01 |
| rs5025718 | 1 | 120478285 | NOTCH2 | C/T | 0.12 | 0.98 | 0.14 |
| rs6695965 | 1 | 147060091 | [BCL9](http://www.ncbi.nlm.nih.gov/entrez/query.fcgi?db=gene&cmd=Retrieve&dopt=Graphics&list_uids=607) | T/G | 0.62 | 0.87 | 0.02 |
| rs2274533 | 1 | 151395782 | POGZ | G/A | 0.93 | 0.48 | 0.17 |
| rs2814778 | 1 | 159174683 | [DARC](http://www.ncbi.nlm.nih.gov/entrez/query.fcgi?db=gene&cmd=Retrieve&dopt=Graphics&list_uids=2532) | A/G | 0.00 | 1.00 | 0.00 |
| rs2065160 | 1 | 204790977 |  | A/G | 0.90 | 0.43 | 0.06 |
| rs6604611 | 1 | 216655251 |  | C/A | 0.09 | 0.88 | 0.06 |
| rs6698938 | 1 | 229985356 |  | A/G | 0.00 | 0.78 | 0.00 |
| rs2502342 | 1 | 243070467 |  | G/A | 0.01 | 0.75 | 0.04 |
| rs883399 | 2 | 9634100 | [ADAM17](http://www.ncbi.nlm.nih.gov/entrez/query.fcgi?db=gene&cmd=Retrieve&dopt=Graphics&list_uids=6868) | A/G | 1.00 | 0.59 | 0.39 |
| rs300152 | 2 | 17986683 |  | T/A | 0.94 | 0.23 | 0.23 |
| rs2384319 | 2 | 26206255 | [KIF3C](http://www.ncbi.nlm.nih.gov/entrez/query.fcgi?db=gene&cmd=Retrieve&dopt=Graphics&list_uids=3797) | G/T | 0.89 | 0.06 | 0.10 |
| rs11124405 | 2 | 35188011 |  | A/G | 0.18 | 0.86 | 0.02 |
| rs13385952 | 2 | 41531398 |  | T/G | 0.06 | 0.94 | 0.22 |
| rs1470524 | 2 | 45129515 |  | G/A | 0.47 | 0.78 | 0.21 |
| rs842634 | 2 | 61091222 | LINC01185 | G/A | 0.77 | 0.03 | 0.26 |
| rs3768641 | 2 | 72368190 | [CYP26B1](http://www.ncbi.nlm.nih.gov/entrez/query.fcgi?db=gene&cmd=Retrieve&dopt=Graphics&list_uids=56603) | G/C | 0.00 | 0.99 | 0.06 |
| rs1881244 | 2 | 73645089 | [ALMS1](http://www.ncbi.nlm.nih.gov/entrez/query.fcgi?db=gene&cmd=Retrieve&dopt=Graphics&list_uids=7840) | G/A | 0.32 | 0.87 | 0.00 |
| rs12714168 | 2 | 86331347 | [POLR1A,PTCD3](http://www.ncbi.nlm.nih.gov/entrez/query.fcgi?db=gene&cmd=Retrieve&dopt=Graphics&list_uids=25885) | T/G | 0.10 | 0.90 | 0.19 |
| rs6576989 | 2 | 97525099 | [ANKRD39, SEMA4C](http://www.ncbi.nlm.nih.gov/entrez/query.fcgi?db=gene&cmd=Retrieve&dopt=Graphics&list_uids=51239) | T/G | 0.00 | 0.91 | 0.07 |
| rs3860446 | 2 | 104489351 |  | G/A | 1.00 | 0.97 | 0.36 |
| rs260714 | 2 | 109562495 | [EDAR](http://www.ncbi.nlm.nih.gov/entrez/query.fcgi?db=gene&cmd=Retrieve&dopt=Graphics&list_uids=10913) | C/T | 0.96 | 0.81 | 0.15 |
| rs951954 | 2 | 110459505 |  | G/A | 0.00 | 0.78 | 0.00 |
| rs901304 | 2 | 163416362 | [KCNH7](http://www.ncbi.nlm.nih.gov/entrez/query.fcgi?db=gene&cmd=Retrieve&dopt=Graphics&list_uids=90134) | T/G | 0.64 | 0.85 | 0.15 |
| rs868179 | 2 | 177549497 |  | G/A | 0.00 | 0.97 | 0.07 |
| rs6748661 | 2 | 195682840 |  | G/A | 0.17 | 0.89 | 0.10 |
| rs10498255 | 2 | 231612230 | CAB39 | A/G | 0.03 | 0.75 | 0.19 |
| rs11713766 | 3 | 398089 | [CHL1](http://www.ncbi.nlm.nih.gov/entrez/query.fcgi?db=gene&cmd=Retrieve&dopt=Graphics&list_uids=10752) | G/A | 0.00 | 0.86 | 0.00 |
| rs2470644 | 3 | 5776857 |  | A/G | 0.17 | 0.80 | 0.07 |
| rs9310888 | 3 | 29286762 |  | G/C | 0.00 | 0.97 | 0.08 |
| rs2197896 | 3 | 30032530 | [RBMS3](http://www.ncbi.nlm.nih.gov/entrez/query.fcgi?db=gene&cmd=Retrieve&dopt=Graphics&list_uids=27303) | T/G | 0.46 | 0.89 | 0.00 |
| rs9311121 | 3 | 35928063 |  | C/T | 0.23 | 0.87 | 0.04 |
| rs13069719 | 3 | 71506545 | [FOXP1](http://www.ncbi.nlm.nih.gov/entrez/query.fcgi?db=gene&cmd=Retrieve&dopt=Graphics&list_uids=27086) | C/T | 0.80 | 0.22 | 0.20 |
| rs2660769 | 3 | 87080496 |  | A/G | 0.44 | 0.99 | 0.17 |
| rs1395771 | 3 | 96463580 |  | G/C | 0.57 | 0.97 | 0.02 |
| rs12489482 | 3 | 104579408 |  | G/A | 0.01 | 0.87 | 0.22 |
| rs6437783 | 3 | 108172817 | [MYH15](http://www.ncbi.nlm.nih.gov/entrez/query.fcgi?db=gene&cmd=Retrieve&dopt=Graphics&list_uids=22989) | T/G | 0.90 | 0.28 | 0.26 |
| rs11714866 | 3 | 110067902 |  | A/G | 0.42 | 0.81 | 0.05 |
| rs6772085 | 3 | 118584563 |  | T/G | 0.00 | 0.81 | 0.09 |
| rs2165139 | 3 | 139214470 |  | A/T | 0.93 | 0.04 | 0.13 |
| rs6439896 | 3 | 139864353 | [CLSTN2](http://www.ncbi.nlm.nih.gov/entrez/query.fcgi?db=gene&cmd=Retrieve&dopt=Graphics&list_uids=64084) | T/G | 0.00 | 0.66 | 0.03 |
| rs1439013 | 3 | 152581991 |  | T/G | 0.26 | 0.74 | 0.01 |
| rs1984473 | 3 | 155811284 |  | G/A | 0.03 | 0.03 | 0.37 |
| rs6804094 | 3 | 187057970 |  | A/T | 0.87 | 0.03 | 0.35 |
| rs1398829 | 4 | 22023275 |  | A/T | 0.00 | 0.03 | 0.02 |
| rs10032047 | 4 | 63727833 |  | G/A | 0.12 | 0.94 | 0.01 |
| rs7689609 | 4 | 72083374 | [SLC4A4](http://www.ncbi.nlm.nih.gov/entrez/query.fcgi?db=gene&cmd=Retrieve&dopt=Graphics&list_uids=8671) | T/G | 0.43 | 1.00 | 0.07 |
| rs6446975 | 4 | 75036044 | [MTHFD2L](http://www.ncbi.nlm.nih.gov/entrez/query.fcgi?db=gene&cmd=Retrieve&dopt=Graphics&list_uids=441024) | G/A | n/a | 0.90 | 0.01 |
| rs7687935 | 4 | 82065566 | [PRKG2](http://www.ncbi.nlm.nih.gov/entrez/query.fcgi?db=gene&cmd=Retrieve&dopt=Graphics&list_uids=5593) | A/T | 0.15 | 0.79 | 0.15 |
| rs7662047 | 4 | 103091730 |  | G/A | 0.00 | 0.89 | 0.01 |
| rs7657799 | 4 | 105375423 |  | G/T | 0.00 | 0.77 | 0.02 |
| rs9307613 | 4 | 130357404 |  | A/T | 0.17 | 0.03 | 0.22 |
| rs13108157 | 4 | 151530763 | [LRBA](http://www.ncbi.nlm.nih.gov/entrez/query.fcgi?db=gene&cmd=Retrieve&dopt=Graphics&list_uids=987) | C/T | 0.10 | 0.94 | 0.10 |
| rs6829588 | 4 | 165221971 | MARCH1 | T/A | 0.30 | 0.81 | 0.05 |
| rs2332031 | 4 | 171742958 |  | T/G | 0.59 | 0.88 | 0.03 |
| rs814597 | 5 | 10468929 |  | C/T | 0.84 | 0.19 | 0.12 |
| rs257748 | 5 | 15819615 | FBXL7 | T/A | 0.96 | 0.97 | 0.38 |
| rs463240 | 5 | 25845146 |  | G/A | 0.00 | 0.77 | 0.02 |
| rs35395 | 5 | 33948589 | [SLC45A2](http://www.ncbi.nlm.nih.gov/entrez/query.fcgi?db=gene&cmd=Retrieve&dopt=Graphics&list_uids=51151) | C/T | 0.95 | 0.79 | 0.00 |
| rs16891982 | 5 | 33951693 | SLC45A2 | G/C | 0.99 | 1.00 | 0.00 |
| rs10059859 | 5 | 59231307 | [PDE4D](http://www.ncbi.nlm.nih.gov/entrez/query.fcgi?db=gene&cmd=Retrieve&dopt=Graphics&list_uids=5144) | T/C | 0.04 | 0.82 | 0.19 |
| rs1443985 | 5 | 119425507 |  | A/G | 0.17 | 0.89 | 0.09 |
| rs10515535 | 5 | 143516142 |  | G/A | 0.13 | 0.97 | 0.29 |
| rs4513684 | 5 | 147652085 | [SPINK13](http://www.ncbi.nlm.nih.gov/entrez/query.fcgi?db=gene&cmd=Retrieve&dopt=Graphics&list_uids=153218) | A/C | 0.03 | 0.81 | 0.00 |
| rs1551765 | 5 | 153176578 | [GRIA1](http://www.ncbi.nlm.nih.gov/entrez/query.fcgi?db=gene&cmd=Retrieve&dopt=Graphics&list_uids=2890) | C/T | 0.88 | 0.29 | 0.19 |
| rs567442 | 5 | 176253285 | [UNC5A](http://www.ncbi.nlm.nih.gov/entrez/query.fcgi?db=gene&cmd=Retrieve&dopt=Graphics&list_uids=90249) | A/T | 0.09 | 0.86 | 0.02 |
| rs6909271 | 6 | 198379 |  | T/A | 0.37 | 0.91 | 0.12 |
| rs6911727 | 6 | 9116398 |  | G/A | 1.00 | 0.97 | 0.48 |
| rs6459548 | 6 | 17482317 | [CAP2](http://www.ncbi.nlm.nih.gov/entrez/query.fcgi?db=gene&cmd=Retrieve&dopt=Graphics&list_uids=10486) | G/A | 0.01 | 0.80 | 0.00 |
| rs10484578 | 6 | 35246319 | ZNF76 | G/A | 0.07 | 0.97 | 0.38 |
| rs1341567 | 6 | 76628176 | [MYO6](http://www.ncbi.nlm.nih.gov/entrez/query.fcgi?db=gene&cmd=Retrieve&dopt=Graphics&list_uids=4646) | G/T | 0.00 | 0.81 | 0.00 |
| rs2497150 | 6 | 84847013 | CEP162, [KIAA1009](http://www.ncbi.nlm.nih.gov/entrez/query.fcgi?db=gene&cmd=Retrieve&dopt=Graphics&list_uids=22832) | C/T | 0.11 | 0.83 | 0.04 |
| rs794672 | 6 | 95458317 |  | G/A | 0.26 | 0.94 | 0.08 |
| rs218867 | 6 | 121398535 |  | A/G | 0.76 | 0.87 | 0.13 |
| rs9320808 | 6 | 121654596 | C6orf170, TBC1D32 | G/A | 0.97 | 0.97 | 0.10 |
| rs6569792 | 6 | 132694751 | MOXD1 | A/G | 0.20 | 0.03 | 0.32 |
| rs6930928 | 6 | 156605556 |  | C/A | 0.07 | 0.84 | 0.03 |
| rs7810554 | 7 | 15141190 |  | A/T | 0.25 | 0.98 | 0.15 |
| rs10486576 | 7 | 28119143 | JAZF1 | A/G | 0.80 | 0.03 | 0.10 |
| rs7784684 | 7 | 40167762 |  | C/A | 0.00 | 0.85 | 0.00 |
| rs10264353 | 7 | 43321077 | HECW1 | A/G | 0.40 | 1.00 | 0.26 |
| rs10214949 | 7 | 79048593 | MAGI2 | A/G | 0.03 | 0.03 | 0.16 |
| rs10257477 | 7 | 107704688 | LAMB4 | A/G | 0.00 | 0.72 | 0.00 |
| rs4727700 | 7 | 107807083 | NRCAM | A/T | n/a | n/a | n/a |
| rs3094537 | 7 | 109554669 |  | A/G | 0.01 | 0.83 | 0.01 |
| rs2021782 | 7 | 132134995 | PLXNA4 | A/G | 0.33 | 0.87 | 0.02 |
| rs10954631 | 7 | 138539626 | KIAA1549 | G/A | 0.01 | 0.84 | 0.10 |
| rs6601288 | 8 | 8943430 |  | T/A | 0.91 | 0.15 | 0.29 |
| rs11778591 | 8 | 12720349 |  | A/C | 0.93 | 0.67 | 0.04 |
| rs9325872 | 8 | 20480271 |  | G/A | 1.00 | 0.94 | 0.32 |
| rs2439522 | 8 | 97533766 | SDC2 | A/G | 0.65 | 0.04 | 0.08 |
| rs4733652 | 8 | 129844527 |  | C/T | 0.83 | 0.97 | 0.24 |
| rs1871534 | 8 | 145639681 | [SLC39A4](http://www.ncbi.nlm.nih.gov/entrez/query.fcgi?db=gene&cmd=Retrieve&dopt=Graphics&list_uids=55630) | C/G | 0.00 | 0.95 | 0.02 |
| rs12347078 | 9 | 344508 | DOCK8 | A/C | 0.01 | 0.89 | 0.02 |
| rs4478653 | 9 | 21853221 | MTAP | A/G | 0.98 | 0.81 | 0.43 |
| rs2482531 | 9 | 25570411 |  | A/C | n/a | n/a | n/a |
| rs4013967 | 9 | 76897070 | MIR6130 | G/A | 0.00 | 0.94 | 0.35 |
| rs10491654 | 9 | 102139527 | NAMA | G/A | 0.90 | 0.50 | 0.29 |
| rs587364 | 9 | 125760863 | RABGAP1 | G/A | 0.72 | 1.00 | 0.13 |
| rs10508349 | 10 | 8298964 |  | G/A | 0.77 | 0.06 | 0.01 |
| rs10748592 | 10 | 94878355 | LOC389997 | C/A | 0.99 | 0.42 | 0.22 |
| rs1572396 | 10 | 117325021 | ATRNL1 | G/A | 0.98 | 0.63 | 0.32 |
| rs1397618 | 10 | 120832675 | EIF3A | A/T | 0.00 | 0.71 | 0.05 |
| rs6485600 | 11 | 12259702 | MICAL2 | G/A | 0.96 | 0.67 | 0.32 |
| rs948360 | 11 | 66106725 | BRMS1 | G/A | 0.00 | 0.75 | 0.03 |
| rs1638567 | 11 | 67125223 | LOC100130987 | A/G | 0.57 | 0.85 | 0.07 |
| rs2458640 | 11 | 78035856 | GAB2 | A/C | 0.75 | 0.94 | 0.22 |
| rs10501474 | 11 | 80400647 |  | C/T | 0.20 | 0.94 | 0.36 |
| rs533571 | 11 | 100850202 | ARHGAP42 | G/A | 0.97 | 0.18 | 0.33 |
| rs4936512 | 11 | 120155336 | POU2F3 | A/G | 0.97 | 0.31 | 0.16 |
| rs1648180 | 11 | 128049402 |  | G/A | 0.97 | 0.45 | 0.22 |
| rs879780 | 11 | 130008104 | APLP2 | T/C | 0.01 | 0.67 | 0.05 |
| rs4762106 | 12 | 66018473 |  | G/C | 0.67 | 0.81 | 0.09 |
| rs4076700 | 12 | 117383320 | FBXW8 | A/G | 0.10 | 0.79 | 0.17 |
| rs2293048 | 12 | 117664825 | NOS1 | G/A | 0.63 | 0.56 | 0.09 |
| rs4034627 | 12 | 128397472 |  | G/A | 0.03 | 0.78 | 0.05 |
| rs2585901 | 13 | 21420271 | XPO4 | G/A | 0.93 | 0.24 | 0.15 |
| rs2065982 | 13 | 34864240 |  | A/G | 0.82 | 0.08 | 0.02 |
| rs1540979 | 13 | 95090692 | DCT | A/T | 0.71 | 0.03 | 0.07 |
| rs10492585 | 13 | 105386176 |  | A/G | 0.00 | 0.90 | 0.06 |
| rs9323178 | 14 | 23113646 |  | G/A | 0.97 | 0.18 | 0.45 |
| rs1451928 | 14 | 48340741 |  | A/C | 0.80 | 0.14 | 0.14 |
| rs10131076 | 14 | 80774385 | DIO2_AS1 | G/A | 0.01 | 0.69 | 0.07 |
| rs730570 | 14 | 101142890 |  | A/G | 0.97 | 0.85 | 0.15 |
| rs2714758 | 15 | 25479337 | SNORD115-35 | A/G | 0.00 | 0.89 | 0.08 |
| rs1129038 | 15 | 28356859 | HERC2 | A/G | 0.99 | 1.00 | 0.22 |
| rs1426654 | 15 | 48426484 | SLC24A5 | A/G | 1.00 | 0.99 | 0.00 |
| rs9302185 | 15 | 54954864 |  | G/C | 0.03 | 0.88 | 0.19 |
| rs10520678 | 15 | 88937283 |  | G/A | 0.00 | 0.85 | 0.27 |
| rs11073967 | 15 | 91565804 | VPS33B | A/G | 1.00 | 0.98 | 0.44 |
| rs9937955 | 16 | 10950526 |  | T/A | 0.90 | 0.57 | 0.17 |
| rs1557519 | 16 | 14251303 | MKL2 | A/G | 0.08 | 0.96 | 0.07 |
| rs30125 | 16 | 14354661 | MKL2 | G/C | 0.10 | 0.64 | 0.05 |
| rs1004704 | 16 | 48537421 |  | G/A | 0.87 | 0.02 | 0.21 |
| rs10500505 | 16 | 64943026 |  | T/A | 0.80 | 0.06 | 0.21 |
| rs4130513 | 16 | 78458750 | WWOX | G/A | 0.19 | 0.72 | 0.08 |
| rs10491097 | 17 | 19361211 |  | G/A | 0.87 | 0.94 | 0.35 |
| rs2253624 | 17 | 69732081 |  | C/A | 0.00 | 0.82 | 0.00 |
| rs1013459 | 18 | 11700534 | GNAL | A/G | 0.03 | 0.77 | 0.10 |
| rs12953952 | 18 | 67737927 | RTTN | G/A | 0.03 | 0.86 | 0.07 |
| rs888861 | 19 | 35381852 | LINC00904 | A/G | 0.10 | n/a | 0.26 |
| rs798887 | 19 | 54793188 |  | A/G | 0.87 | 0.22 | 0.15 |
| rs708915 | 20 | 8400667 | PLCB1 | T/A | 0.73 | 0.74 | 0.10 |
| rs2208139 | 20 | 37908954 |  | A/G | 0.97 | 0.17 | 0.33 |
| rs2829454 | 21 | 26273071 | LOC339622 | G/C | 0.93 | 0.06 | 0.27 |
| rs138022 | 22 | 40613036 | TNRC6B | G/A | 0.03 | 0.83 | 0.26 |
| rs992864 | X | 110500583 | CAPN6 | G/A | 0.00 | 0.95 | 0.04 |
| rs1867024 | X | 147980413 | [AFF2](http://www.ncbi.nlm.nih.gov/entrez/query.fcgi?db=gene&cmd=Retrieve&dopt=Graphics&list_uids=2334) | A/G | 0.04 | 0.94 | 0.06 |

^a^Chromosomal position from assembly GRCh37/hg19.

^b^Gene information obtained with SNP nexus (http://www.snp-nexus.org/).

^c^Allele frequencies in the parental populations correspond to those of the European minor allele.

**Table S2.** Primers and restriction sites used for mitochondrial DNA haplogroup assignment.

| **haplogroup** | **forward primer** | **reverse primer** | **restriction site** | **reference** |
| --- | --- | --- | --- | --- |
| A | 612 | 742 | HaeIII (+) | Martínez-Cruzado et al. (2005) |
| B | 8216 | 8296 | none | Martínez-Cruzado et al. (2005) |
| D | 5121 | 5229 | 5176 AluI (-) | Martínez-Cruzado et al. (2005) |
| C | 13233 | 13809 | 13259 HincII (-) | Martínez-Cruzado et al. (2005) |
| H | 6958 | 7261 | 7025 AluI (-) | Martínez-Cruzado et al. (2005)(F); Bai et al. (2007)(R) |
| V | 4462 | 4919 | 4577 NlaIII (-) | Martínez-Cruzado et al. (2005) |
| U | 12265 | 12308 | 12308 HinfI (+) | Martínez-Cruzado et al. (2005) |
| J/T | 4160 | 4489 | 4216 NlaIII (+) | Martínez-Cruzado et al. (2005)(F); Bai et al. (2007)(R) |
| T | 13233 | 13809 | 13366 DpnII (+) | Martínez-Cruzado et al. (2005) |
| K | 8921 | 9086 | 9055 HhaI (-) | Martínez-Cruzado et al. (2005) |
| L | 10814 | 10912 | 10873 MnlI (-) | Sans et al. (2011) |

**Table S3.** Confounders and nuclear individual ancestry in breast cancer cases and controls from Uruguay.

|  | **% African**  **(mean ± SD)** | **% Native American**  **(mean ± SD)** | **% European**  **(mean ± SD)** |
| --- | --- | --- | --- |
| **controls (N=164)** |  |  |  |
| **location** **(N)** |  |  |  |
| urban (145) | 9.6 ± 7.7 | 14.6 ± 11.1 | 75.8 ± 14.0 |
| rural (15) | 6.2 ± 4.8 | 14.7 ± 12.4 | 79.1 ± 14.3 |
| p-value | 0.14 | 0.88 | 0.33 |
| **oral contraceptives (N)** |  |  |  |
| yes (121) | 9.3 ± 7.7 | 15.0 ± 11.6 | 75.7 ± 14.3 |
| no (43) | 8.6 ± 6.7 | 13.7 ± 10.1 | 77.7 ± 13.9 |
| p-value | 0.78 | 0.71 | 0.39 |
| **children (N)** |  |  |  |
| yes (150) | 9.3 ± 7.7 | 14.6 ± 11.1 | 76.1 ± 14.2 |
| no (14) | 7.2 ± 4.6 | 16.0 ± 13.0 | 76.9 ± 14.0 |
| p-value | 0.57 | 0.76 | 0.81 |
| **breastfeeding (N)** |  |  |  |
| yes (130) | 9.5 ± 7.9 | 15.2 ± 11.3 | 75.3 ± 14.5 |
| no (22) | 8.9 ± 5.8 | 15.3 ± 12.3 | 75.9 ± 13.1 |
| p-value | 0.88 | 0.90 | 0.96 |
| **smoking (N)** |  |  |  |
| yes (86) | 9.9 ± 8.6 | 14.9 ± 11.4 | 75.2 ± 15.2 |
| no (75) | 8.5 ± 6.0 | 14.3 ± 11.2 | 77.2 ± 13.1 |
| p-value | 0.56 | 0.68 | 0.51 |
| **passive smoker (N)** |  |  |  |
| yes (82) | 9.7 ± 7.7 | 15.5 ± 12.0 | 74.8 ± 15.2 |
| no (70) | 9.2 ± 7.6 | 14.9 ± 10.7 | 76.0 ± 13.0 |
| p-value | 0.71 | 0.90 | 0.76 |
| **occupation** **(N)** |  |  |  |
| unemployed/never worked (1) | 2.3 | 24.9 | 72.8 |
| self-employed (112) | 10.9 ± 7.9 | 16.6 ± 11.7 | 72.5 ± 14.4 |
| public/private employee (16) | 5.3 ± 4.3 | 12.6 ± 10.4 | 82.1 ± 9.2 |
| teacher (7) | 6.9 ± 7.2 | 10.0 ± 10.2 | 83.1 ± 16.0 |
| retired (7) | 5.9 ± 5.1 | 12.4 ± 8.2 | 81.6 ± 8.8 |
| professional (17) | 5.7 ± 4.1 | 7.6 ± 7.4 | 86.7 ± 7.8 |
| p-value | 0.01 | 0.02 | 3x10^-4^ |
| **income** **(U$S per month, N)** |  |  |  |
| < 175 (11) | 6.8 ± 5.0 | 17.4 ± 11.0 | 75.9 ± 12.2 |
| 175-750 (104) | 10.1 ± 8.3 | 16.2 ± 11.6 | 73.7 ± 14.6 |
| >750 (41) | 7.6 ± 5.5 | 10.1 ± 9.9 | 82.3 ± 12.0 |
| p-value | 0.11 | 0.01 | 0.004 |
| **source of income (N)** |  |  |  |
| social security/family (6) | 14.9 ± 5.8 | 22.3 ± 11.0 | 62.8 ± 14.3 |
| pension (7) | 5.2 ± 1.9 | 8.7 ± 5.2 | 86.1 ± 6.3 |
| salary (143) | 9.3 ± 7.6 | 14.8 ± 11.5 | 75.9 ± 14.2 |
| rent (2) | 2.5 ± 0.1 | 14.5 ± 9.1 | 83.2 ± 9.1 |
| other (3) | 10.8 ± 8.1 | 10.9 ± 4.2 | 78.2 ± 12.0 |
| p-value | 0.12 | 0.29 | 0.05 |
| **age at menarche** (mean change in years per 25% ancestry increase, 95% CI) (N=161) | 0.03 (-0.16, 0.22) | 0.15 (-0.05,0.34) | -0.11 (-0.31, 0.08) |
| p-value | 0.78 | 0.14 | 0.25 |
| **number of children** (mean change per 25% ancestry increase, 95% CI) (N=152) | 0.18 (-0.06, 0.41) | -0.19 (-0.43, 0.05) | -0.02 (-0.04, 0.004) |
| p-value | 0.34 | 0.12 | 0.11 |
| **weight** (mean change in kg per 25% ancestry increase, 95% CI) (N=160) | 0.43 (-1.40, 2.26) | 0.84 (-1.05, 2.72) | -0.92 (-2.76, 0.93) |
| p-value | 0.64 | 0.38 | 0.33 |
| **height** (mean change in cm per 25% ancestry increase, 95% CI) (N=161) | 0.67 (-0.24, 1.58) | -1.29 (-2.22, -0.36) | 0.44 (-0.48, 1.36) |
| p-value | 0.15 | 0.01 | 0.35 |
| **BMI** (mean change in kg/m^2^ per 25% ancestry increase, 95% CI) (N=160) | -0.07 (-0.77, 0.63) | 0.76 (0.05, 1.47) | -0.52 (-1.22, 0.18) |
| p-value | 0.84 | 0.04 | 0.15 |
| **waist-hip ratio** (mean change in ratio per 25% ancestry increase, 95% CI) (N=135) | -0.01 (-0.02, 0.01) | -0.002 (-0.01, 0.01) | 0.01 (-0.004, 0.02) |
| p-value | 0.36 | 0.71 | 0.22 |

**Table S4.** Association of ancestry informative markers with breast cancer risk. Results obtained using the Cochran-Armitage trend test.

| **SNP** | **chr** | **chr position(bp)** | **unadjusted p-value** | **genomic control p-value** | **Bonferroni correction p-value** |
| --- | --- | --- | --- | --- | --- |
| rs424436 | 1 | 8082678 | 0.928 | 0.928 | 1 |
| rs2817611 | 1 | 11613163 | 0.826 | 0.826 | 1 |
| rs6684063 | 1 | 30699340 | 0.598 | 0.598 | 1 |
| rs1931059 | 1 | 35366056 | 0.822 | 0.823 | 1 |
| rs10908316 | 1 | 35494810 | 0.731 | 0.731 | 1 |
| rs2791966 | 1 | 36314861 | 0.029 | 0.030 | 1 |
| rs710232 | 1 | 42203154 | 0.127 | 0.129 | 1 |
| rs1934393 | 1 | 49208618 | 0.797 | 0.797 | 1 |
| rs596985 | 1 | 64277035 | 0.489 | 0.490 | 1 |
| rs855833 | 1 | 64357432 | 0.130 | 0.132 | 1 |
| rs3828121 | 1 | 82422200 | 0.870 | 0.870 | 1 |
| rs17035850 | 1 | 117032022 | 0.146 | 0.147 | 1 |
| rs5025718 | 1 | 120478285 | 0.345 | 0.347 | 1 |
| rs6695965 | 1 | 147060091 | 0.245 | 0.247 | 1 |
| rs2274533 | 1 | 151395782 | 0.712 | 0.713 | 1 |
| rs2814778 | 1 | 159174683 | 0.146 | 0.147 | 1 |
| rs2065160 | 1 | 204790977 | 0.281 | 0.282 | 1 |
| rs6604611 | 1 | 216655251 | 0.809 | 0.809 | 1 |
| rs6698938 | 1 | 229985356 | 0.312 | 0.314 | 1 |
| rs2502342 | 1 | 243070467 | 0.183 | 0.184 | 1 |
| rs883399 | 2 | 9634100 | 0.449 | 0.450 | 1 |
| rs300152 | 2 | 17986683 | 0.591 | 0.592 | 1 |
| rs2384319 | 2 | 26206255 | 0.654 | 0.655 | 1 |
| rs11124405 | 2 | 35188011 | 0.285 | 0.287 | 1 |
| rs13385952 | 2 | 41531398 | 0.359 | 0.361 | 1 |
| rs1470524 | 2 | 45129515 | 0.501 | 0.501 | 1 |
| rs842634 | 2 | 61091222 | 0.104 | 0.104 | 1 |
| rs3768641 | 2 | 72368190 | 0.724 | 0.725 | 1 |
| rs1881244 | 2 | 73645089 | 0.329 | 0.331 | 1 |
| rs12714168 | 2 | 86331347 | 0.905 | 0.905 | 1 |
| rs2660769 | 2 | 87080496 | 0.989 | 0.990 | 1 |
| rs6576989 | 2 | 97525099 | 0.986 | 0.986 | 1 |
| rs3860446 | 2 | 104489351 | 0.031 | 0.031 | 1 |
| rs260714 | 2 | 109562495 | 0.021 | 0.021 | 1 |
| rs951954 | 2 | 110459505 | 0.349 | 0.351 | 1 |
| rs901304 | 2 | 163416362 | 0.822 | 0.823 | 1 |
| rs868179 | 2 | 177549497 | 0.941 | 0.941 | 1 |
| rs6748661 | 2 | 195682840 | 0.441 | 0.443 | 1 |
| rs10498255 | 2 | 231612230 | 0.828 | 0.828 | 1 |
| rs11713766 | 3 | 398089 | 0.597 | 0.598 | 1 |
| rs2470644 | 3 | 5776857 | 0.025 | 0.025 | 1 |
| rs9310888 | 3 | 29286762 | 0.645 | 0.645 | 1 |
| rs2197896 | 3 | 30032530 | 0.082 | 0.084 | 1 |
| rs9311121 | 3 | 35928063 | 0.272 | 0.274 | 1 |
| rs13069719 | 3 | 71506545 | 0.223 | 0.225 | 1 |
| rs1395771 | 3 | 96463580 | 0.082 | 0.082 | 1 |
| rs12489482 | 3 | 104579408 | 0.894 | 0.895 | 1 |
| rs6437783 | 3 | 108172817 | 0.693 | 0.694 | 1 |
| rs11714866 | 3 | 110067902 | 0.933 | 0.933 | 1 |
| rs6772085 | 3 | 118584563 | 0.052 | 0.053 | 1 |
| rs2165139 | 3 | 139214470 | 0.477 | 0.479 | 1 |
| rs6439896 | 3 | 139864353 | 0.758 | 0.759 | 1 |
| rs1439013 | 3 | 152581991 | 0.691 | 0.692 | 1 |
| rs1984473 | 3 | 155811284 | 0.779 | 0.779 | 1 |
| rs6804094 | 3 | 187057970 | 0.076 | 0.076 | 1 |
| rs1398829 | 4 | 22023275 | 0.951 | 0.951 | 1 |
| rs10032047 | 4 | 63727833 | 0.687 | 0.688 | 1 |
| rs7689609 | 4 | 72083374 | 0.818 | 0.818 | 1 |
| rs6446975 | 4 | 75036044 | 0.864 | 0.865 | 1 |
| rs7687935 | 4 | 82065566 | 0.048 | 0.049 | 1 |
| rs7662047 | 4 | 103091730 | 0.430 | 0.432 | 1 |
| rs7657799 | 4 | 105375423 | 0.739 | 0.740 | 1 |
| rs9307613 | 4 | 130357404 | 0.840 | 0.840 | 1 |
| rs13108157 | 4 | 151530763 | 0.841 | 0.841 | 1 |
| rs6829588 | 4 | 165221971 | 0.271 | 0.273 | 1 |
| rs2332031 | 4 | 171742958 | 0.029 | 0.029 | 1 |
| rs814597 | 5 | 10468929 | 0.032 | 0.033 | 1 |
| rs257748 | 5 | 15819615 | 0.173 | 0.173 | 1 |
| rs463240 | 5 | 25845146 | 0.894 | 0.894 | 1 |
| rs16891982 | 5 | 33951693 | 0.699 | 0.700 | 1 |
| rs10059859 | 5 | 59231307 | 0.239 | 0.241 | 1 |
| rs1443985 | 5 | 119425507 | 0.260 | 0.262 | 1 |
| rs10515535 | 5 | 143516142 | 0.050 | 0.050 | 1 |
| rs4513684 | 5 | 147652085 | 0.864 | 0.865 | 1 |
| rs1551765 | 5 | 153176578 | 0.364 | 0.366 | 1 |
| rs567442 | 5 | 176253285 | 0.199 | 0.201 | 1 |
| rs6909271 | 6 | 198379 | 0.561 | 0.562 | 1 |
| rs6911727 | 6 | 9116398 | 0.597 | 0.597 | 1 |
| rs6459548 | 6 | 17482317 | 0.597 | 0.598 | 1 |
| rs10484578 | 6 | 35246319 | 0.594 | 0.594 | 1 |
| rs2497150 | 6 | 84847013 | 0.783 | 0.784 | 1 |
| rs794672 | 6 | 95458317 | 0.355 | 0.356 | 1 |
| rs218867 | 6 | 121398535 | 0.877 | 0.878 | 1 |
| rs9320808 | 6 | 121654596 | 0.788 | 0.788 | 1 |
| rs6569792 | 6 | 132694751 | 0.453 | 0.453 | 1 |
| rs6930928 | 6 | 156605556 | 0.684 | 0.685 | 1 |
| rs7810554 | 7 | 15141190 | 0.742 | 0.743 | 1 |
| rs10486576 | 7 | 28119143 | 0.027 | 0.027 | 1 |
| rs7784684 | 7 | 40167762 | 0.975 | 0.975 | 1 |
| rs10264353 | 7 | 43321077 | 0.046 | 0.047 | 1 |
| rs10214949 | 7 | 79048593 | 0.655 | 0.655 | 1 |
| rs10257477 | 7 | 107704688 | 0.796 | 0.796 | 1 |
| rs4727700 | 7 | 107807083 | 0.672 | 0.673 | 1 |
| rs3094537 | 7 | 109554669 | 0.032 | 0.033 | 1 |
| rs2021782 | 7 | 132134995 | 0.743 | 0.743 | 1 |
| rs10954631 | 7 | 138539626 | 0.314 | 0.316 | 1 |
| rs6601288 | 8 | 8943430 | 0.151 | 0.152 | 1 |
| rs11778591 | 8 | 12720349 | 0.416 | 0.417 | 1 |
| rs9325872 | 8 | 20480271 | 0.835 | 0.835 | 1 |
| rs2439522 | 8 | 97533766 | 0.572 | 0.573 | 1 |
| rs4733652 | 8 | 129844527 | 0.191 | 0.191 | 1 |
| rs1871534 | 8 | 145639681 | 0.994 | 0.994 | 1 |
| rs12347078 | 9 | 344508 | 0.046 | 0.047 | 1 |
| rs4478653 | 9 | 21853221 | 0.457 | 0.458 | 1 |
| rs2482531 | 9 | 25570411 | 0.317 | 0.319 | 1 |
| rs4013967 | 9 | 76897070 | 0.635 | 0.635 | 1 |
| rs10491654 | 9 | 102139527 | 0.977 | 0.977 | 1 |
| rs587364 | 9 | 125760863 | 0.600 | 0.601 | 1 |
| rs10508349 | 10 | 8298964 | 0.676 | 0.676 | 1 |
| rs10748592 | 10 | 94878355 | 0.486 | 0.487 | 1 |
| rs1572396 | 10 | 117325021 | 0.609 | 0.610 | 1 |
| rs1397618 | 10 | 120832675 | 0.567 | 0.567 | 1 |
| rs6485600 | 11 | 12259702 | 0.603 | 0.604 | 1 |
| rs948360 | 11 | 66106725 | 0.484 | 0.484 | 1 |
| rs1638567 | 11 | 67125223 | 0.692 | 0.693 | 1 |
| rs2458640 | 11 | 78035856 | 0.776 | 0.777 | 1 |
| rs10501474 | 11 | 80400647 | 0.418 | 0.418 | 1 |
| rs533571 | 11 | 100850202 | 0.960 | 0.961 | 1 |
| rs4936512 | 11 | 120155336 | 0.498 | 0.500 | 1 |
| rs1648180 | 11 | 128049402 | 0.095 | 0.096 | 1 |
| rs879780 | 11 | 130008104 | 0.065 | 0.065 | 1 |
| rs4762106 | 12 | 66018473 | 0.931 | 0.931 | 1 |
| rs4076700 | 12 | 117383320 | 0.996 | 0.996 | 1 |
| rs2293048 | 12 | 117664825 | 0.201 | 0.203 | 1 |
| rs4034627 | 12 | 128397472 | 0.975 | 0.975 | 1 |
| rs2585901 | 13 | 21420271 | 0.019 | 0.019 | 1 |
| rs2065982 | 13 | 34864240 | 0.085 | 0.086 | 1 |
| rs1540979 | 13 | 95090692 | 0.117 | 0.118 | 1 |
| rs10492585 | 13 | 105386176 | 0.402 | 0.402 | 1 |
| rs9323178 | 14 | 23113646 | 1.000 | 1.000 | 1 |
| rs1451928 | 14 | 48340741 | 0.945 | 0.945 | 1 |
| rs10131076 | 14 | 80774385 | 0.819 | 0.819 | 1 |
| rs730570 | 14 | 101142890 | 0.562 | 0.563 | 1 |
| rs2714758 | 15 | 25479337 | 0.035 | 0.036 | 1 |
| rs1129038 | 15 | 28356859 | 0.872 | 0.873 | 1 |
| rs1426654 | 15 | 48426484 | 0.698 | 0.699 | 1 |
| rs9302185 | 15 | 54954864 | 0.492 | 0.492 | 1 |
| rs10520678 | 15 | 88937283 | 0.777 | 0.777 | 1 |
| rs11073967 | 15 | 91565804 | 0.886 | 0.887 | 1 |
| rs9937955 | 16 | 10950526 | 0.724 | 0.725 | 1 |
| rs1557519 | 16 | 14251303 | 0.570 | 0.571 | 1 |
| rs30125 | 16 | 14354661 | 0.852 | 0.852 | 1 |
| rs1004704 | 16 | 48537421 | 0.072 | 0.072 | 1 |
| rs10500505 | 16 | 64943026 | 0.085 | 0.085 | 1 |
| rs4130513 | 16 | 78458750 | 0.704 | 0.704 | 1 |
| rs10491097 | 17 | 19361211 | 0.850 | 0.850 | 1 |
| rs2253624 | 17 | 69732081 | 0.246 | 0.246 | 1 |
| rs1013459 | 18 | 11700534 | 0.076 | 0.076 | 1 |
| rs12953952 | 18 | 67737927 | 0.727 | 0.727 | 1 |
| rs888861 | 19 | 35381852 | 0.495 | 0.495 | 1 |
| rs798887 | 19 | 54793188 | 0.886 | 0.886 | 1 |
| rs708915 | 20 | 8400667 | 0.454 | 0.454 | 1 |
| rs2208139 | 20 | 37908954 | 0.040 | 0.040 | 1 |
| rs2829454 | 21 | 26273071 | 0.530 | 0.530 | 1 |
| rs138022 | 22 | 40613036 | 0.436 | 0.436 | 1 |
| rs992864 | X | 110500583 | 0.845 | 0.845 | 1 |
| rs1867024 | X | 147980413 | 0.196 | 0.197 | 1 |

Note: rs7504, rs1341567 and rs35395 were not included in the analysis because they were out of Hardy-Weinberg equilibrium.

**Table S5.** Association of ancestry informative markers with breast cancer risk. Results obtained using logistic regression with adjustment for European and Native American ancestry.

| **SNP** | **chr** | **chr position (bp)** | **effect allele** | **OR** | **95% CI** | **p-value** |
| --- | --- | --- | --- | --- | --- | --- |
| rs424436 | 1 | 8082678 | C | 1.43 | (0.57,3.57) | 0.449 |
| rs2817611 | 1 | 11613163 | A | 1.06 | (0.56,2.01) | 0.861 |
| rs6684063 | 1 | 30699340 | G | 1.02 | (0.67,1.56) | 0.913 |
| rs1931059 | 1 | 35366056 | A | 1.08 | (0.62,1.90) | 0.779 |
| rs10908316 | 1 | 35494810 | G | 0.73 | (0.37,1.46) | 0.375 |
| rs2791966 | 1 | 36314861 | T | 0.44 | (0.24,0.81) | 0.008 |
| rs710232 | 1 | 42203154 | C | 0.52 | (0.19,1.38) | 0.187 |
| rs1934393 | 1 | 49208618 | G | 1.08 | (0.74,1.57) | 0.691 |
| rs596985 | 1 | 64277035 | C | 1.98 | (0.50,7.79) | 0.331 |
| rs855833 | 1 | 64357432 | C | 0.59 | (0.30,1.18) | 0.136 |
| rs3828121 | 1 | 82422200 | C | 1.23 | (0.78,1.94) | 0.371 |
| rs17035850 | 1 | 117032022 | T | 2.76 | (0.57,13.44) | 0.210 |
| rs5025718 | 1 | 120478285 | T | 1.57 | (0.74,3.33) | 0.241 |
| rs6695965 | 1 | 147060091 | C | 1.61 | (0.86,3.01) | 0.138 |
| rs2274533 | 1 | 151395782 | T | 1.43 | (0.73,2.77) | 0.294 |
| rs2814778 | 1 | 159174683 | G | 2.85 | (0.63,12.96) | 0.174 |
| rs2065160 | 1 | 204790977 | C | 0.84 | (0.43,1.66) | 0.624 |
| rs6604611 | 1 | 216655251 | A | 0.90 | (0.47,1.73) | 0.753 |
| rs6698938 | 1 | 229985356 | G | 0.41 | (0.12,1.40) | 0.157 |
| rs2502342 | 1 | 243070467 | T | 1.82 | (0.68,4.89) | 0.237 |
| rs883399 | 2 | 9634100 | G | 0.98 | (0.63,1.53) | 0.923 |
| rs300152 | 2 | 17986683 | T | 0.95 | (0.57,1.58) | 0.838 |
| rs2384319 | 2 | 26206255 | G | 0.90 | (0.49,1.67) | 0.747 |
| rs11124405 | 2 | 35188011 | G | 0.77 | (0.32,1.90) | 0.575 |
| rs13385952 | 2 | 41531398 | C | 1.32 | (0.70,2.49) | 0.384 |
| rs1470524 | 2 | 45129515 | T | 0.95 | (0.65,1.38) | 0.770 |
| rs842634 | 2 | 61091222 | T | 0.75 | (0.48,1.16) | 0.195 |
| rs3768641 | 2 | 72368190 | C | 1.22 | (0.59,2.53) | 0.600 |
| rs1881244 | 2 | 73645089 | A | 0.75 | (0.36,1.53) | 0.424 |
| rs12714168 | 2 | 86331347 | C | 0.94 | (0.50,1.74) | 0.835 |
| rs2660769 | 2 | 87080496 | G | 1.03 | (0.60,1.77) | 0.924 |
| rs6576989 | 2 | 97525099 | C | 0.95 | (0.44,2.05) | 0.896 |
| rs3860446 | 2 | 104489351 | T | 0.70 | (0.50,1.00) | 0.048 |
| rs260714 | 2 | 109562495 | T | 0.59 | (0.33,1.04) | 0.067 |
| rs951954 | 2 | 110459505 | A | 0.63 | (0.15,2.66) | 0.528 |
| rs901304 | 2 | 163416362 | C | 0.99 | (0.55,1.79) | 0.980 |
| rs868179 | 2 | 177549497 | A | 1.02 | (0.55,1.88) | 0.959 |
| rs6748661 | 2 | 195682840 | A | 1.26 | (0.62,2.56) | 0.531 |
| rs10498255 | 2 | 231612230 | C | 1.00 | (0.68,1.45) | 0.980 |
| rs11713766 | 3 | 398089 | A | 0.81 | (0.19,3.38) | 0.773 |
| rs2470644 | 3 | 5776857 | G | 4.33 | (1.46,12.84) | 0.008 |
| rs9310888 | 3 | 29286762 | G | 1.06 | (0.56,1.98) | 0.868 |
| rs2197896 | 3 | 30032530 | C | 2.83 | (1.22,6.57) | 0.016 |
| rs9311121 | 3 | 35928063 | T | 1.58 | (0.74,3.38) | 0.239 |
| rs13069719 | 3 | 71506545 | T | 0.81 | (0.46,1.40) | 0.443 |
| rs1395771 | 3 | 96463580 | G | 0.70 | (0.40,1.24) | 0.222 |
| rs12489482 | 3 | 104579408 | A | 1.13 | (0.60,2.12) | 0.712 |
| rs6437783 | 3 | 108172817 | C | 0.83 | (0.49,1.41) | 0.486 |
| rs11714866 | 3 | 110067902 | G | 1.17 | (0.62,2.21) | 0.629 |
| rs6772085 | 3 | 118584563 | C | 2.49 | (1.03,5.99) | 0.042 |
| rs2165139 | 3 | 139214470 | T | 0.92 | (0.40,2.13) | 0.851 |
| rs6439896 | 3 | 139864353 | C | 0.81 | (0.26,2.52) | 0.715 |
| rs1439013 | 3 | 152581991 | C | 1.66 | (0.79,3.46) | 0.182 |
| rs1984473 | 3 | 155811284 | T | 0.98 | (0.70,1.36) | 0.882 |
| rs6804094 | 3 | 187057970 | T | 1.60 | (1.09,2.34) | 0.017 |
| rs1398829 | 4 | 22023275 | T | 1.04 | (0.52,2.08) | 0.922 |
| rs10032047 | 4 | 63727833 | A | 0.97 | (0.46,2.06) | 0.937 |
| rs7689609 | 4 | 72083374 | C | 1.44 | (0.77,2.67) | 0.251 |
| rs6446975 | 4 | 75036044 | A | 0.93 | (0.36,2.38) | 0.883 |
| rs7687935 | 4 | 82065566 | T | 0.53 | (0.30,0.91) | 0.023 |
| rs7662047 | 4 | 103091730 | A | 1.41 | (0.54,3.65) | 0.480 |
| rs7657799 | 4 | 105375423 | G | 1.77 | (0.51,6.10) | 0.364 |
| rs9307613 | 4 | 130357404 | A | 1.08 | (0.77,1.53) | 0.654 |
| rs13108157 | 4 | 151530763 | T | 1.23 | (0.56,2.68) | 0.611 |
| rs6829588 | 4 | 165221971 | T | 0.76 | (0.41,1.40) | 0.376 |
| rs2332031 | 4 | 171742958 | C | 0.57 | (0.31,1.04) | 0.065 |
| rs814597 | 5 | 10468929 | T | 3.44 | (1.42,8.33) | 0.006 |
| rs257748 | 5 | 15819615 | A | 0.82 | (0.58,1.15) | 0.249 |
| rs463240 | 5 | 25845146 | A | 0.88 | (0.39,2.01) | 0.763 |
| rs16891982 | 5 | 33951693 | C | 1.10 | (0.62,1.98) | 0.741 |
| rs10059859 | 5 | 59231307 | T | 1.44 | (0.69,3.03) | 0.335 |
| rs1443985 | 5 | 119425507 | G | 0.79 | (0.45,1.40) | 0.423 |
| rs10515535 | 5 | 143516142 | A | 0.74 | (0.53,1.03) | 0.076 |
| rs4513684 | 5 | 147652085 | C | 0.87 | (0.34,2.22) | 0.776 |
| rs1551765 | 5 | 153176578 | T | 1.44 | (0.79,2.62) | 0.230 |
| rs567442 | 5 | 176253285 | T | 0.69 | (0.34,1.40) | 0.306 |
| rs6909271 | 6 | 198379 | A | 1.10 | (0.61,1.96) | 0.756 |
| rs6911727 | 6 | 9116398 | C | 0.84 | (0.60,1.18) | 0.307 |
| rs6459548 | 6 | 17482317 | A | 0.72 | (0.20,2.55) | 0.606 |
| rs10484578 | 6 | 35246319 | A | 0.94 | (0.66,1.32) | 0.708 |
| rs2497150 | 6 | 84847013 | T | 1.01 | (0.46,2.18) | 0.989 |
| rs794672 | 6 | 95458317 | A | 0.75 | (0.39,1.45) | 0.393 |
| rs218867 | 6 | 121398535 | G | 1.09 | (0.62,1.94) | 0.760 |
| rs9320808 | 6 | 121654596 | A | 1.13 | (0.75,1.73) | 0.556 |
| rs6569792 | 6 | 132694751 | G | 1.08 | (0.76,1.54) | 0.657 |
| rs6930928 | 6 | 156605556 | A | 1.17 | (0.53,2.61) | 0.701 |
| rs7810554 | 7 | 15141190 | T | 0.91 | (0.51,1.61) | 0.736 |
| rs10486576 | 7 | 28119143 | C | 1.97 | (1.23,3.13) | 0.005 |
| rs7784684 | 7 | 40167762 | T | 0.78 | (0.18,3.39) | 0.737 |
| rs10264353 | 7 | 43321077 | G | 0.69 | (0.40,1.20) | 0.191 |
| rs10214949 | 7 | 79048593 | G | 1.11 | (0.67,1.84) | 0.686 |
| rs10257477 | 7 | 107704688 | C | 1.23 | (0.49,3.09) | 0.661 |
| rs4727700 | 7 | 107807083 | T | 1.18 | (0.69,2.03) | 0.551 |
| rs3094537 | 7 | 109554669 | C | 6.60 | (1.33,32.83) | 0.021 |
| rs2021782 | 7 | 132134995 | G | 1.25 | (0.68,2.32) | 0.475 |
| rs10954631 | 7 | 138539626 | A | 1.45 | (0.68,3.09) | 0.331 |
| rs6601288 | 8 | 8943430 | A | 0.79 | (0.49,1.27) | 0.332 |
| rs11778591 | 8 | 12720349 | C | 1.07 | (0.58,1.97) | 0.820 |
| rs9325872 | 8 | 20480271 | A | 0.99 | (0.71,1.40) | 0.962 |
| rs2439522 | 8 | 97533766 | G | 1.41 | (0.65,3.05) | 0.384 |
| rs4733652 | 8 | 129844527 | T | 0.81 | (0.52,1.25) | 0.338 |
| rs1871534 | 8 | 145639681 | G | 1.10 | (0.39,3.11) | 0.864 |
| rs12347078 | 9 | 344508 | C | 0.37 | (0.14,0.98) | 0.045 |
| rs4478653 | 9 | 21853221 | C | 0.80 | (0.50,1.28) | 0.349 |
| rs2482531 | 9 | 25570411 | C | 1.21 | (0.68,2.16) | 0.525 |
| rs4013967 | 9 | 76897070 | T | 0.91 | (0.63,1.32) | 0.625 |
| rs10491654 | 9 | 102139527 | T | 1.04 | (0.74,1.47) | 0.806 |
| rs587364 | 9 | 125760863 | T | 1.02 | (0.56,1.86) | 0.939 |
| rs10508349 | 10 | 8298964 | A | 1.14 | (0.54,2.38) | 0.733 |
| rs10748592 | 10 | 94878355 | T | 0.93 | (0.55,1.55) | 0.771 |
| rs1572396 | 10 | 117325021 | A | 0.99 | (0.58,1.69) | 0.972 |
| rs1397618 | 10 | 120832675 | T | 1.43 | (0.70,2.92) | 0.327 |
| rs6485600 | 11 | 12259702 | A | 0.87 | (0.53,1.44) | 0.585 |
| rs948360 | 11 | 66106725 | A | 1.16 | (0.67,2.02) | 0.594 |
| rs1638567 | 11 | 67125223 | C | 1.02 | (0.48,2.17) | 0.955 |
| rs2458640 | 11 | 78035856 | C | 0.99 | (0.57,1.72) | 0.968 |
| rs10501474 | 11 | 80400647 | T | 1.18 | (0.83,1.70) | 0.357 |
| rs533571 | 11 | 100850202 | A | 1.13 | (0.68,1.88) | 0.640 |
| rs4936512 | 11 | 120155336 | C | 1.01 | (0.58,1.78) | 0.962 |
| rs1648180 | 11 | 128049402 | A | 0.75 | (0.46,1.22) | 0.250 |
| rs879780 | 11 | 130008104 | C | 0.61 | (0.38,0.98) | 0.039 |
| rs4762106 | 12 | 66018473 | G | 1.20 | (0.81,1.78) | 0.357 |
| rs4076700 | 12 | 117383320 | C | 0.98 | (0.64,1.50) | 0.911 |
| rs2293048 | 12 | 117664825 | T | 0.76 | (0.41,1.40) | 0.379 |
| rs4034627 | 12 | 128397472 | T | 1.12 | (0.58,2.17) | 0.737 |
| rs2585901 | 13 | 21420271 | T | 0.67 | (0.45,1.02) | 0.059 |
| rs2065982 | 13 | 34864240 | C | 0.65 | (0.32,1.31) | 0.226 |
| rs1540979 | 13 | 95090692 | T | 1.65 | (0.91,3.00) | 0.098 |
| rs10492585 | 13 | 105386176 | C | 1.21 | (0.71,2.06) | 0.475 |
| rs9323178 | 14 | 23113646 | G | 1.00 | (0.72,1.38) | 0.995 |
| rs1451928 | 14 | 48340741 | G | 1.09 | (0.74,1.61) | 0.650 |
| rs10131076 | 14 | 80774385 | A | 1.09 | (0.62,1.90) | 0.763 |
| rs730570 | 14 | 101142890 | G | 1.58 | (0.88,2.82) | 0.125 |
| rs2714758 | 15 | 25479337 | G | 4.82 | (1.29,17.98) | 0.019 |
| rs1129038 | 15 | 28356859 | A | 1.02 | (0.61,1.69) | 0.948 |
| rs1426654 | 15 | 48426484 | G | 1.13 | (0.53,2.44) | 0.751 |
| rs9302185 | 15 | 54954864 | C | 1.13 | (0.70,1.83) | 0.626 |
| rs10520678 | 15 | 88937283 | T | 1.05 | (0.69,1.58) | 0.828 |
| rs11073967 | 15 | 91565804 | A | 0.89 | (0.53,1.48) | 0.646 |
| rs9937955 | 16 | 10950526 | A | 0.96 | (0.54,1.68) | 0.874 |
| rs1557519 | 16 | 14251303 | C | 0.73 | (0.35,1.54) | 0.406 |
| rs30125 | 16 | 14354661 | G | 0.88 | (0.50,1.55) | 0.650 |
| rs1004704 | 16 | 48537421 | A | 0.75 | (0.51,1.09) | 0.127 |
| rs10500505 | 16 | 64943026 | A | 0.82 | (0.53,1.27) | 0.374 |
| rs4130513 | 16 | 78458750 | T | 0.93 | (0.55,1.58) | 0.796 |
| rs10491097 | 17 | 19361211 | A | 1.02 | (0.70,1.47) | 0.935 |
| rs2253624 | 17 | 69732081 | T | 1.76 | (0.82,3.77) | 0.150 |
| rs1013459 | 18 | 11700534 | G | 1.60 | (0.98,2.62) | 0.059 |
| rs12953952 | 18 | 67737927 | A | 1.02 | (0.55,1.86) | 0.961 |
| rs888861 | 19 | 35381852 | G | 0.86 | (0.61,1.22) | 0.401 |
| rs798887 | 19 | 54793188 | G | 1.12 | (0.79,1.61) | 0.520 |
| rs708915 | 20 | 8400667 | A | 0.96 | (0.67,1.38) | 0.832 |
| rs2208139 | 20 | 37908954 | C | 0.72 | (0.51,1.03) | 0.070 |
| rs2829454 | 21 | 26273071 | G | 1.14 | (0.79,1.62) | 0.488 |
| rs138022 | 22 | 40613036 | A | 0.82 | (0.54,1.24) | 0.340 |
| rs992864 | X | 110500583 | A | 0.78 | (0.35,1.77) | 0.557 |
| rs1867024 | X | 147980413 | G | 0.67 | (0.29,1.60) | 0.371 |

Note: rs7504, rs1341567 and rs35395 were not included in the analysis because they were out of Hardy-Weinberg equilibrium.

**Table S6.** Potential confounders and mitochondrial DNA ancestry in Uruguayan controls.

|  | **African (%)^a^** | **Native American (%)^a^** | **European (%)^a^** |
| --- | --- | --- | --- |
| **controls (N=164)**^b^ | 16 | 65 | 83 |
| **place of residence (N)** |  |  |  |
| Montevideo (143) | 81.3 | 96.9 | 80.7 |
| outside Montevideo (21) | 18.7 | 3.1 | 19.3 |
| p-value | 0.01 |  |  |
| **location (N)** |  |  |  |
| urban (145) | 93.8 | 95.3 | 86.3 |
| rural (15) | 6.2 | 4.7 | 13.8 |
| p-value | 0.16 |  |  |
| **hospital (N)** |  |  |  |
| public (139) | 81.3 | 93.9 | 78.3 |
| private (25) | 18.7 | 6.1 | 21.7 |
| p-value | 0.02 |  |  |
| **oral contraceptives (N)** |  |  |  |
| yes (121) | 68.9 | 72.3 | 75.9 |
| no (43) | 31.2 | 27.7 | 24.1 |
| p-value | 0.76 |  |  |
| **children (N)** |  |  |  |
| yes (150) | 93.8 | 93.9 | 89.2 |
| no (14) | 6.2 | 6.1 | 10.8 |
| p-value | 0.66 |  |  |
| **breastfeeding (N)** |  |  |  |
| yes (130) | 87.5 | 88.5 | 82.7 |
| no (22) | 12.5 | 11.5 | 17.3 |
| p-value | 0.67 |  |  |
| **menopause (N)** |  |  |  |
| yes (91) | 60.0 | 56.9 | 56.3 |
| no (69) | 40.0 | 43.1 | 43.8 |
| p-value | 1.00 |  |  |
| **breast cancer in the family (N)** |  |  |  |
| yes (16) | 0.0 | 10.8 | 10.8 |
| no (148) | 100.0 | 89.2 | 89.2 |
| p-value | 0.58 |  |  |
| **smoking (N)** |  |  |  |
| yes (86) | 62.5 | 59.4 | 46.9 |
| no (75) | 37.5 | 40.6 | 53.1 |
| p-value | 0.25 |  |  |
| **passive smoker (N)** |  |  |  |
| yes (82) | 43.8 | 55.7 | 54.7 |
| no (70) | 56.3 | 44.3 | 45.3 |
| p-value | 0.69 |  |  |
| **education (N)** |  |  |  |
| primary school (53) | 31.3 | 35.4 | 30.1 |
| secondary school (71) | 31.3 | 47.7 | 42.2 |
| university (40) | 37.5 | 16.9 | 27.7 |
| p-value | 0.37 |  |  |
| **occupation (N)** |  |  |  |
| unemployed/never worked (1) | 0.0 | 1.5 | 0.0 |
| self-employed (112) | 66.7 | 78.4 | 63.7 |
| public/private employee (16) | 0.0 | 10.8 | 11.2 |
| teacher (7) | 13.3 | 3.1 | 3.8 |
| retired (7) | 6.7 | 3.1 | 5.0 |
| professional (17) | 13.3 | 3.1 | 16.3 |
| p-value | 0.09 |  |  |
| **income (N)** |  |  |  |
| < 175 (11) | 0.0 | 4.7 | 10.4 |
| 175-750 (104) | 62.5 | 79.4 | 57.1 |
| >750 (41) | 37.5 | 15.9 | 32.5 |
| p-value | 0.04 |  |  |
| **source of income (N)** |  |  |  |
| social security/family (6) | 0.0 | 4.6 | 3.8 |
| pension (7) | 0.0 | 1.5 | 7.5 |
| salary (143) | 87.5 | 90.8 | 87.5 |
| rent (2) | 6.3 | 0.0 | 1.2 |
| other (3) | 6.3 | 3.1 | 0.0 |
| p-value | 0.13 |  |  |
| **socioeconomic status (N)** |  |  |  |
| low (53) | 25.0 | 35.4 | 31.3 |
| medium (111) | 75.0 | 64.6 | 68.7 |
| p-value | 0.71 |  |  |
| **age at recruitment (mean ± SD)** | 52.6 ± 8.6 | 53.0 ± 8.3 | 52.7 ± 9.6 |
| p-value | 0.97 |  |  |
| **age at menarche (mean ± SD) (N=161)** | 12.9 ± 1.3 | 12.1 ± 1.6 | 12.2 ± 1.3 |
| p-value | 0.12 |  |  |
| **age at first child (mean ± SD) (N=139)** | 24.5 ± 5.4 | 22.4 ± 6.3 | 24.0 ± 5.5 |
| p-value | 0.25 |  |  |
| **number of children** **(mean ± SD) (N=152)** | 2.3 ± 1.9 | 2.7 ± 1.9 | 2.4 ± 1.5 |
| p-value | 0.62 |  |  |
| **age at menopause (mean ± SD) (N=49)^c^** | 47.8 ± 6.9 | 47.5 ± 4.7 | 47.6 ± 3.4 |
| p-value | 0.99 |  |  |
| **weight** **(kg mean ± SD)** | 66.1 ± 12.4 | 68.2 ± 11.5 | 69.3 ± 15.2 |
| p-value | 0.66 |  |  |
| **height** **(cm mean ± SD) (N=161)** | 161.4 ± 4.8 | 158.0 ± 6.7 | 160 ± 7.1 |
| p-value | 0.09 |  |  |
| **BMI** **(mean ± SD)** | 25.4 ± 4.8 | 27.4 ± 4.6 | 27.0 ± 5.6 |
| p-value | 0.39 |  |  |
| **waist-hip ratio** **(mean ± SD) (N=135)** | 0.84 ± 0.07 | 0.86 ± 0.07 | 0.86 ± 0.09 |
| p-value | 0.72 |  |  |

^a^Percentage of individuals with an African, Native American or European mitochondrial haplogroup who are included in each category of the confounder.

^b^Fisher’s exact test was run for all categorical confounders.

^c^Age at menopause was calculated for women who were ≥ 55 years old and had undergone menopause naturally.
